# Recent Advances in Inverse-Electron-Demand Hetero-Diels–Alder Reactions of 1-Oxa-1,3-Butadienes

**DOI:** 10.1007/s41061-016-0026-2

**Published:** 2016-04-20

**Authors:** Aleksandra Pałasz

**Affiliations:** Department of Organic Chemistry, Jagiellonian University, Ingardena 3 St, 30-060 Kraków, Poland

**Keywords:** Hetero-Diels–Alder reactions, 1-Oxa-1,3-butadienes, Dihydropyrans, Domino Knoevenagel hetero-Diels–Alder reactions, Bioorthogonal cycloaddition

## Abstract

This review is an endeavor to highlight the progress in the inverse-electron-demand hetero-Diels–Alder reactions of 1-oxa-1,3-butadienes in recent years. The huge number of examples of 1-oxadienes cycloadditions found in the literature clearly demonstrates the incessant importance of this transformation in pyran ring synthesis. This type of reaction is today one of the most important methods for the synthesis of dihydropyrans which are the key building blocks in structuring of carbohydrate and other natural products. Two different modes, inter- and intramolecular, of inverse-electron-demand hetero-Diels–Alder reactions of 1-oxadienes are discussed. The domino Knoevenagel hetero-Diels–Alder reactions are also described. In recent years the use of chiral Lewis acids, chiral organocatalysts, new optically active heterodienes or dienophiles have provided enormous progress in asymmetric synthesis. Solvent-free and aqueous hetero-Diels–Alder reactions of 1-oxabutadienes were also investigated. The reactivity of reactants, selectivity of cycloadditions, and chemical stability in aqueous solutions and under physiological conditions were taken into account to show the potential application of the described reactions in bioorthogonal chemistry. New bioorthogonal ligation by click inverse-electron-demand hetero-Diels–Alder cycloaddition of in situ-generated 1-oxa-1,3-butadienes and vinyl ethers was developed. It seems that some of the hetero-Diels–Alder reactions described in this review can be applied in bioorthogonal chemistry because they are selective, non-toxic, and can function in biological conditions taking into account pH, an aqueous environment, and temperature.

## Introduction

Cycloaddition reactions provide quick and economic methods for the construction of monocyclic, polycyclic and heterocyclic systems. The use of hetero-substituted diene and dienophiles is important for the application of Diels–Alder cycloadditions towards natural and biologically active product synthesis. Dihydro- and tetrahydropyran derivatives are prevalent structural subunits in a variety of natural compounds, including carbohydrates, pheromones, alkaloids, iridoids and polyether antibiotics [1–8]. The abundance of carbohydrates in living cells is a reason for the development of new synthetic procedures for the preparation of natural and unnatural sugars. There are two synthetic routes leading to dihydropyran derivatives via [4 + 2] cycloadditions. The first one is the [4 + 2] cycloaddition of the carbonyl group of aldehydes or ketones, acting as heterodienophiles, with electron-rich 1,3-butadienes [9–23]. The second route is the hetero-Diels–Alder (HDA) reactions of electron-deficient α,β-unsaturated carbonyl compounds, representing an 1-oxa-1,3-butadiene system, with electron-rich alkenes. Excellent diastereoselectivity is a characteristic feature of heterocycloaddition of many substituted α,β-unsaturated carbonyl compounds. The HDA reactions of oxabutadienes also show a high regioselectivity. These reactions have been classified as cycloadditions with inverse-electron-demand [24]. The reviews on this topic have already been published but they cover the literature until only 1997 [1–8, 24]. The most comprehensive one was written by Tietze and Kettschau in Topics in Current Chemistry in 1997 [2]. The presented review is an endeavor to highlight the progress in the HDA reactions with inverse-electron-demand of 1-oxa-1,3-butadienes after the year 2000.

The reactivity of α,β-unsaturated carbonyl compounds in HDA reactions is low and the reactions must be conducted at high temperature [25–27] or under high pressure [28–30]. The use of enol ethers as dienophiles with electron-donating groups improves the cycloadditions but high temperature is needed and diastereoselectivity of these reactions is still low. Aza-substituted dienophiles have been used more rarely than their oxygenated counterparts in the HDA reactions of 1-oxa-1,3-butadienes. Enamines can participate in these reactions, providing entry to highly complex molecules [31–33]. The reactivity of 1-oxa-1,3-butadiene can be enhanced by introducing electron-withdrawing substituents [34–39]. Presence of an electron-withdrawing group in the 1-oxadiene system lowers the lowest energy unoccupied molecular orbital (LUMO) energy level which then can more easily overlap with the highest energy unoccupied molecular orbital (HOMO) orbital of the dienophile. Tietze et al. calculated the influence of various substituents on the energy of LUMO orbitals in 4-*N*-acetylamino-1-oxa-1,3-butadienes using semiempirical methods [40]. It was found that the energy depends on the type and position of a substituent in the 1-oxadiene system. The cyano and trifluoromethyl groups in the 3 position were found to have the highest influence on reactivity of 1-oxa-1,3-butadienes in cycloadditions with enol ethers. In addition to the effect of the substituents in the heterodiene, Lewis acid catalysts, such as ZnCl_2_, TiCl_4_, SnCl_4_, EtAlCl_2_, Me_2_AlCl, LiClO_4_, Mg(ClO_4_)_2_, Eu(fod)_3_, Yb(fod)_3_, accelerate the HDA reactions [41–53]. The choice of the Lewis acid also has influence on the stereoselectivity of cycloadditions because this catalyst is involved in an *endo* or an *exo*-transition structure and steric interactions are important for stereochemistry.

**Fig. 1:**
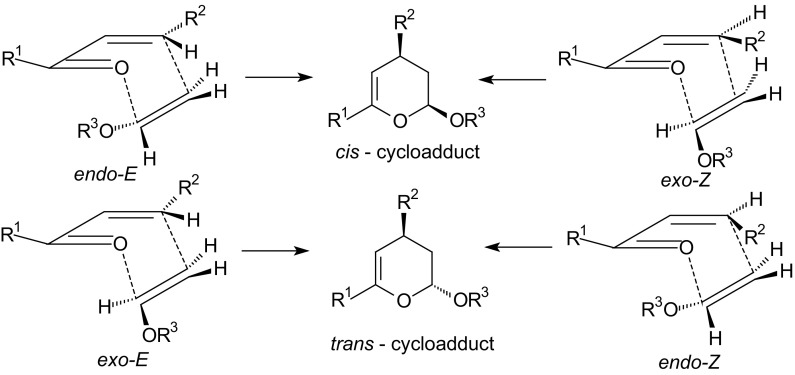
The four different diastereoselective approaches of an alkene such as vinyl ether to 1-oxa-1,3-butadiene for intermolecular HDA reaction

**Fig. 2:**
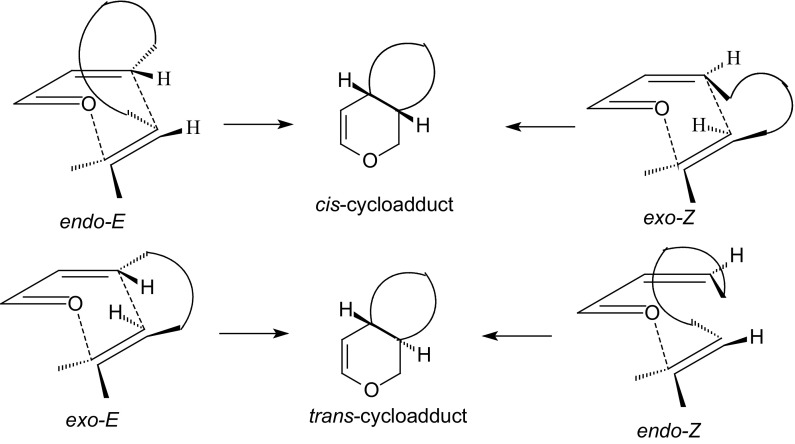
The four different diastereoselective approaches of an alkene to 1-oxa-1,3-butadiene for intramolecular HDA reaction

Inverse-electron-demand HDA reaction between α,β-unsaturated carbonyl compounds and electron-rich alkenes gives an enantioselective approach to chiral dihydropyrans which are precursors for the synthesis of carbohydrate derivatives. To obtain optically active carbohydrate derivatives by the HDA approach, either a chiral transformation via the use of a chiral auxiliary or a catalytic enantioselective reaction is necessary [50–53]. Two different modes of inverse-electron-demand HDA reactions of 1-oxa-1,3-butadienes are discussed in this paper: inter- and intramolecular mode. The geometry of the transition structures of HDA reactions influences the diastereoselectivity of cycloadditions. There are four different transition states for HDA reactions of 1-oxa-1,3-butadienes, according to an *endo*- or *exo*-orientation of the dienophile and an (*E*)- or (*Z*)-configuration of the 1-oxa-1,3-butadiene [2]. The four transition structures for inter- and intramolecular HDA reactions providing the two diastereomers *cis* and *trans* are showed in Figs. 1 and 2. The orientation of the dienophile–vinyl ether, with the alkoxy group being close to the oxygen atom in the heterodiene is called *endo* (Fig. 1) [2]. The opposite is called *exo*. For intramolecular HDA reactions of 1-oxa-1,3-butadienes, the orientation with the chain connecting the heterodiene and dienophile lying close to the heterodiene is called *endo* (Figure 2) [2]. The *cis*-adduct can be formed by an *endo*-*E* or *exo*-*Z* orientation. The *trans*-adduct is obtained by either an *exo*-*E* or *endo*-*Z* transition state (Figs. 1, 2).

Tietze et al. extensively described the domino Knoevenagel hetero-Diels–Alder reactions of unsaturated aromatic and aliphatic aldehydes with different 1,3-dicarbonyl compounds for the synthesis of heterocycles with a pyran ring [54–68]. In the intramolecular mode, the 1-oxa-1,3-butadienes are prepared in situ by a Knoevenagel condensation of aldehydes bearing the dienophile moiety. This method has a broad scope since a multitude of different aldehydes and 1,3-dicarbonyl compounds can be used.

Different examples of inter- and intramolecular HDA reactions of 1-oxa-1,3-butadienes described in literature after the year 2000 are discussed below. The usefulness of HDA reactions of oxadienes is connected with the number of bonds which are formed in one sequence and with the fact that complex molecules can be obtained by this method. Thus, the HDA reactions of α,β-unsaturated carbonyl compounds are atom economic and they allow for regio-, diastereo- and enantioselective synthesis of multifunctional pyran derivatives from relatively simple compounds. Therefore, these cycloadditions can be potentially applied in bioorthogonal chemistry.

## Inverse-Electron-Demand Hetero-Diels–Alder Reactions of 1-Oxa-1,3-Butadienes

### Intermolecular Hetero-Diels–Alder Reactions of 1-Oxa-1,3-Butadienes

#### Non-catalytic Intermolecular Hetero-Diels–Alder Reactions of 1-Oxa-1,3-Butadienes

**Scheme 1:**
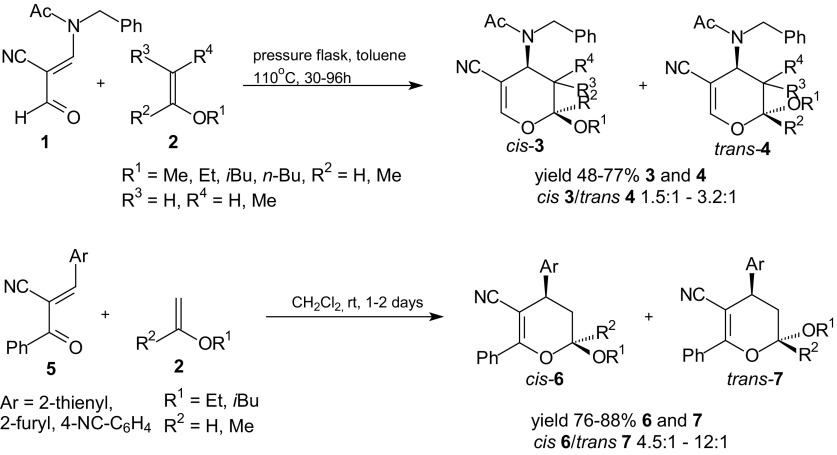
Inverse-electron-demand HDA reactions of propenenitriles **1** and **5** with enol ethers **2**

The reactivity of heterodienes in inverse-electron-demand HDA reactions can be enhanced by introducing electron-withdrawing substituents into the 1-oxa-1,3-butadiene system [34–39, 69–74]. For activation of an oxabutadiene in heterocycloadditions, a cyano group can serve especially well. Such examples are cycloadditions between propenenitriles with a cyano group at C-3 of the heterodiene system [71–74]. Moreover, two papers [71, 72] describe examples of cycloaddition reaction of enaminocarbaldehydes or enaminoketones with enol ethers, leading to 4-amino-3,4-dihydro-2*H*-pyrans. 4-Amino-pyrans are precursors in synthesis of 3-amino sugar derivatives which are present in various antibiotics such as gentamycin C or adriamycin. The HDA reactions of 3-(*N*-acetyl-*N*-benzylamino)-2-formylprop-2-enenitriles **1** with enol ethers **2** yielded *cis* **3** and *trans* **4** diastereoisomers of 2-alkoxy-4-amino-3,4-dihydro-2*H*-pyran-5-carbonitriles in moderate yields (Scheme 1) [71]. The reactions of 2-benzoyl-3-heteroaromaticprop-2-enenitriles **5** with enol ethers **2** afforded diastereoisomeric *cis* **6** and *trans* **7** cycloadducts [71].

Enaminocarbaldehyde **1** was found to be less reactive than propenenitriles **5** since reactions of **5** with enol ethers **2** occurred at room temperature whereas reactions with **1** required heating in boiling toluene.

**Scheme 2:**
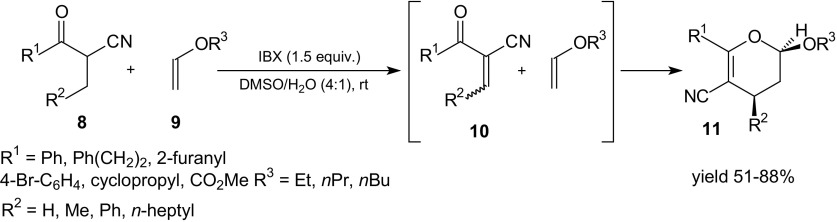
One-pot procedure for the conversion of β-oxonitriles **8** into dihydropyrans **11** by dehydrogenation and HDA reaction

Another interesting example of HDA reaction of 1-oxa-1,3-butadienes with vinyl ethers was described by Klahn and Kirsch [75]. They examined dehydrogenation of β-oxonitriles **8** by treatment with *o*-iodoxybenzoic acid (IBX) at room temperature (Scheme 2). Products of the dehydrogenation–unsaturated counterparts **10** can react in situ, undergoing rapid HDA reactions with enol ethers **9** to produce poly-functionalized dihydropyrans **11**. Cycloadducts **11** were generated in moderate to good yields and with excellent *cis*-diastereoselectivity (up to >99:1).

**Scheme 3:**
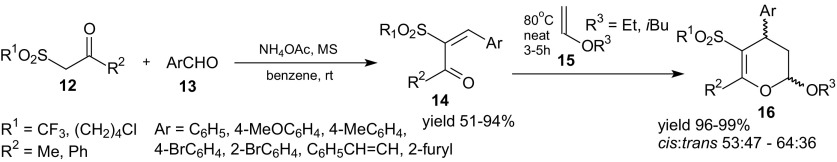
HDA reactions of (*E*)-α-perfluoroalkanesulfonyl-α,β-unsatutated ketones **14**

Xing et al. described cycloadditions of fluorine-containing α,β-unsaturated ketones **14**, which are electron-poor 1-oxa-1,3-butadienes, with electron-rich olefins **15** (Scheme 3) [76]. The ketones **14** were prepared by the Knoevenagel reactions of β-keto perfluoroalkanesulfones **12** with aromatic aldehydes **13** in presence of ammonium acetate as catalyst.

Tetrasubstituted dihydropyrans **16** were prepared in quantitative yields. All the products **16** were the diastereomeric mixtures.

**Scheme 4:**
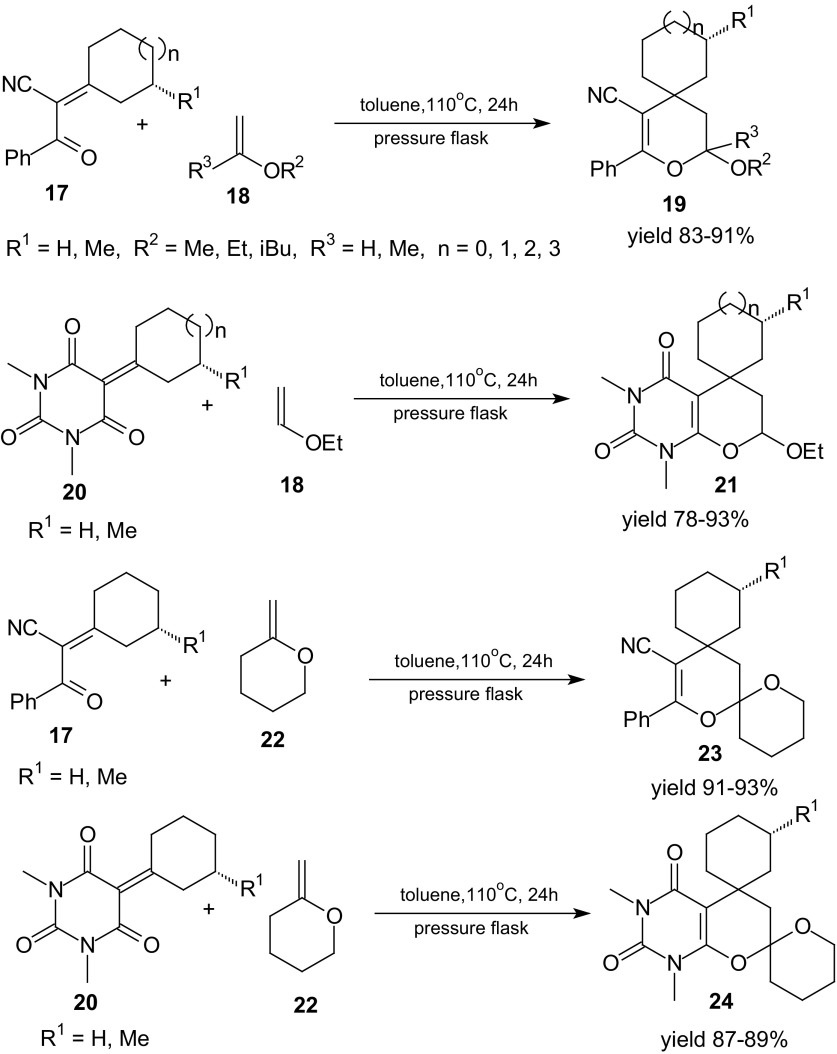
HDA reactions of sterically hindered 2-cycloalkylidene-3-oxo-3-phenylpropionitriles **17** or 5-cycloalkylidene-1,3-dimethylpyrimidine-2,4,6-triones **20** with enol ethers **18** and cyclic enol ether **22**

Another example of HDA reaction of 1-oxa-1,3-butadienes is inverse-electron-demand Diels–Alder cycloaddition of sterically hindered cycloalkylidene derivatives of benzoylacetonitrile **17** and derivatives of *N*,*N*-dimethylbarbituric acid **20** with enol ethers **18** and cyclic enol ether **22** (Scheme 4) [77]. Spirodihydropyrans **19**, dispirodihydropyrans **23**, spirouracils **21**, and dispirouracils **24** were prepared. The cycloaddition reactions of 2-cycloalkylidene-3-oxo-3-phenylpropionitriles **17** or 5-cycloalkylidene-1,3-dimethylpyrimidine-2,4,6-triones **20** with enol ethers **18** were performed in toluene solution at reflux and the pyrans **19** and **21** were obtained in good (78–93 %) yields (Scheme 4). The inverse-electron-demand HDA reactions between cycloalkylidene derivatives **17** or **20** and cyclic enol ether **22** were performed in toluene solution at 110 °C for 24 h and the dispiropyrans **23** and **24** were obtained in good (87–93 %) yields. For all cycloadditions, high diastereoselectivity was observed. Products were each obtained as one enantiomerically pure diastereoisomer. Confirmation of the experimental results by semi-empirical AM1, PM3 methods and ab initio Hartree–Fock calculations of frontier molecular orbital energies of heterodienes (H) and dienophiles (D) has been performed. For reaction of ethyl-vinyl ether, the energy gaps E_LUMO_(H)–E_HOMO_(D) are slightly lower for the cycloalkylidene derivatives of *N*,*N*-dimethylbarbituric acid than for the cycloalkylidene derivatives of benzoylacetonitrile. The reactivity of cyclic enol ether is comparable with the reactivity of ethyl-vinyl ether [77].

**Scheme 5:**
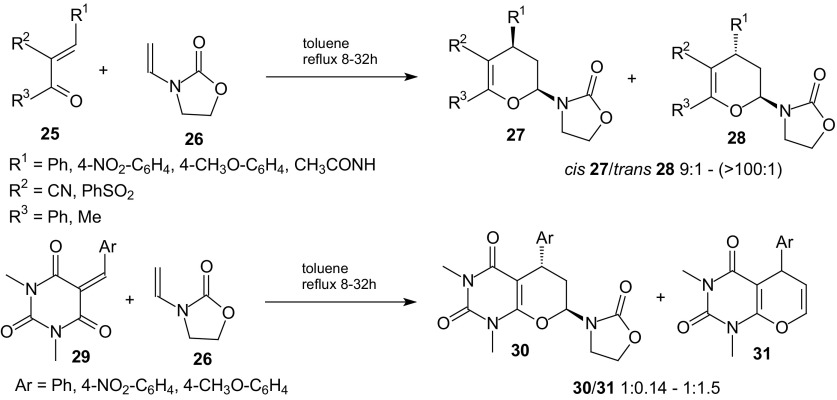
Inverse-electron-demand HDA reactions of different 1-oxa-1,3-butadienes **25** and **29** with *N*-vinyl-2-oxazolidinone **26**

The scope of intermolecular HDA reactions of 1-oxa-1,3-butadienes with inverse-electron-demand was expanded to cycloadditions with enecarbamate [78]. Cycloadditions of 3-aryl-2-benzoylprop-2-enenitriles and 3-phenylsulfonylbut-3-en-2-ones **25** to *N*-vinyl-2-oxazolidinone **26** proceeded regio- and diastereoselectively, yielding *cis* **27** and *trans* **28** diastereoisomers of 3,4-dihydro-2-(2-oxo-3-oxazolidinyl)-2*H*-pyrans in 37–65 % yield (Scheme 5). Cycloadducts *cis*-**27** were the major products. Reactions of 5-arylidene-1,3-dimethylbarbituric acids **29** with dienophile **26** afforded mixtures of pyrano[2,3-*d*]pyrimidinediones *trans* **30** in 50–52 % yield and products **31** resulted from an elimination of 2-oxazolidinone.

*N*-Vinyl-2-oxazolidinone **26** can act as a valuable dienophile in inverse-electron-demand heterocycloaddition. This compound was found to be less reactive than enol ethers because similar reactions of dienes **25** and **29** with enol ethers occurred at room temperature [71, 82] whereas reactions with **26** required heating in boiling toluene.

#### Three-Component Domino Knoevenagel Hetero-Diels–Alder Reactions of 1-Oxa-1,3-Butadienes

**Scheme 6:**
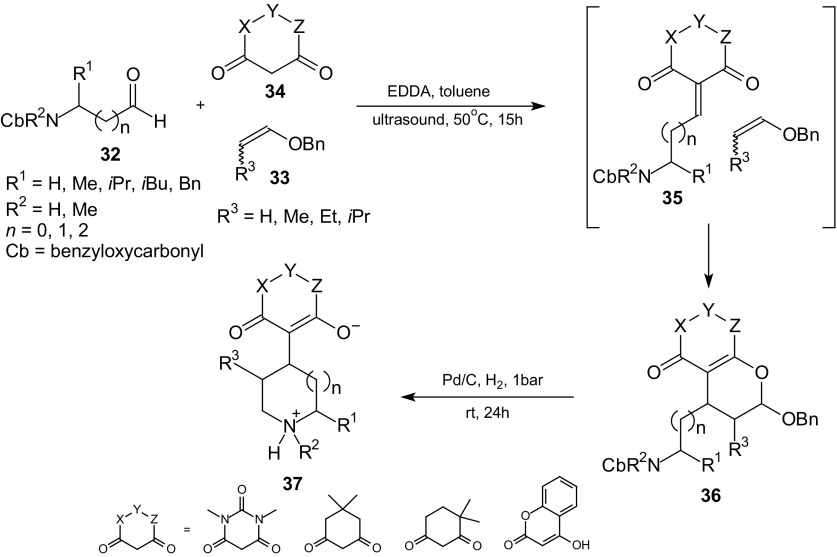
Domino sequence comprising Knoevenagel, inverse-electron-demand HDA reaction and hydrogenation starting from amino aldehydes **32**, 1,3-dicarbonyl compounds **34**, and enol ethers **33**

Domino Knoevenagel hetero-Diels–Alder reactions with an intermolecular cycloaddition can be performed as a three-component reaction using a mixture of a 1,3-dicarbonyl compound, an aldehyde, and a vinyl ether or an enamine. Any cyclic 1,3-dicarbonyl compounds such as 1,3-cyclohexanediones, Meldrum’s acid or *N,N*-dimethylbarbituric acid, as well as reactive acyclic 1,3-dicarbonyl compounds, can be employed. Tietze et al. examined the multicomponent domino Knoevenagel HDA reactions of 1,3-dicarbonyl compounds **34** with amino aldehydes **32** and enol ethers **33**, followed by a reductive amination with the formation of betaines **37** which can be precipitated from the solution in high purity (Scheme 6) [79, 80].

The amino aldehydes **32** were treated with the 1,3-dicarbonyl components **34** and benzoyl enol ethers **33** in toluene in the presence of catalytic amounts of EDDA and trimethyl orthoformate as dehydrating agent in an ultrasonic bath. The domino reaction sequence of Knoevenagel, HDA reaction, and hydrogenation allows rapid access to a number of *N*-heterocycles of different ring sizes and with different substituents in a betaine **37**.

Radi et al. described a protocol for the multicomponent microwave-assisted organocatalytic domino Knoevenagel HDA reaction for the synthesis of substituted 2,3-dihydropyran[2,3-*c*]pyrazoles [81]. The reported procedure can be used for the fast generation of pyran[2,3-*c*]pyrazoles with potential anti-tuberculosis activity.

**Scheme 7:**
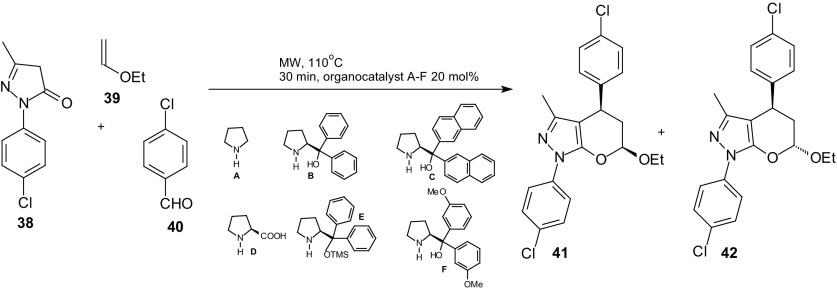
Microwave-assisted organocatalytic mulicomponent Knoevenagel HDA reaction

A mixture of pyrazolone **38**, aldehyde **40** and 10 equiv of ethyl-vinyl ether **39** was MW irradiated and heated at 110 °C in the presence of the appropriate organocatalyst **A**–**F** (Scheme 7). The best results were obtained in the presence of diaryl-prolinols **B** and **C**. In the absence of the catalyst the reaction did not start at all. Using the catalyst **B** and *t*-BuOH as the solvent, the authors obtained the cycloadducts **41** and **42** in yields (56 and 12 %, respectively) and improved diastereoisomeric ratio (4:1) in comparison to the results previously obtained.

**Scheme 8:**
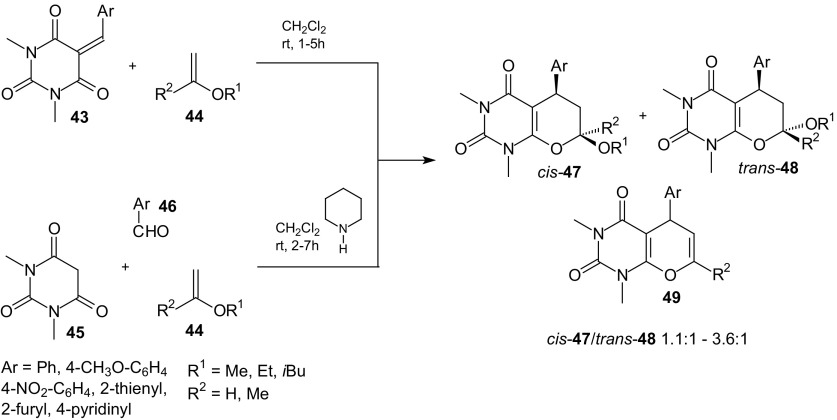
Inverse-electron-demand HDA reactions of 5-arylidene-*N*,*N*-dimethylbarbituric acids **43** with enol ethers **44**. Three-component one-pot synthesis of fused uracils – pyrano[2,3-*d*]-pyrimidine-2,4-diones *cis*-**47** and *trans*-**48**

Inverse-electron-demand HDA reaction of 1-oxa-1,3-butadienes was used in synthesis of the fused uracils–pyrano[2,3-*d*]pyrimidine-2,4-diones [82]. This group of uracils, as a fused heterobicyclic system, constitutes an important contribution in medicinal chemistry and a wide variety of attractive pharmacological effects has been attributed to them [83]. First, it was examined that 5-arylidene-*N*,*N*-dimethylbarbituric acids **43** undergo smooth HDA reactions with enol ethers **44** to afford *cis*-**47** and *trans*-**48** diastereoisomers of 7-alkoxy-5-aryl-2*H*-pyrano[2,3-*d*]pyrimidine-2,4-diones in excellent yields (84–95 %; Scheme 8). Cycloadducts **47** with *cis*-configurations were the major products. Next, three-component one-pot reactions of *N*,*N*-dimethylbarbituric acid **45**, aromatic and heteroaromatic aldehydes **46**, and enol ethers **44** in the presence of piperidine gave uracils *cis*-**47** and *trans*-**48** also in very good yields (87–95 %; Scheme 8). Trace amounts of compounds **49** created by a *trans*-diaxial-elimination of the appropriate alcohol were also obtained in these reactions.

**Scheme 9:**
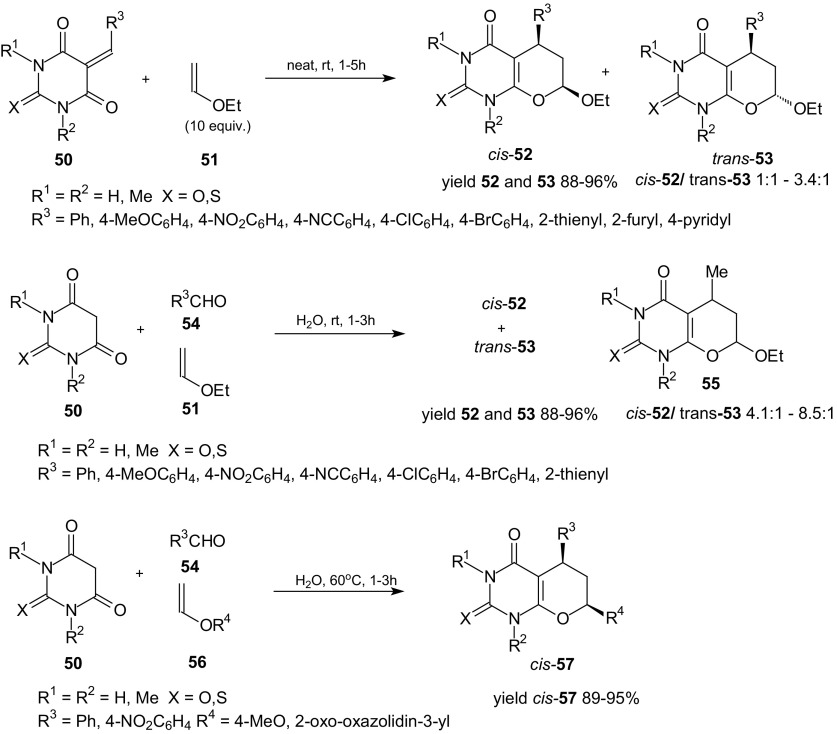
Solvent free HDA reactions of 5-arylidene-*N*,*N*-dimethylbarbituric acids **50** with ethyl vinyl ether **51**. Three-component **50**, **54** and **51** or **56** one-pot synthesis of fused uracils in water

The advantages of these reactions are: the excellent yields, short reactions times, and the fact that cycloadditions do not require drastic conditions, but can be carried out at room temperature. The described reactions give easy and rapid access to both *cis*-**47** as *trans*-**48** diastereoisomers of uracils and pure diastereoisomers can be very easily isolated by column chromatography. Also, solvent-free HDA reactions of 5-arylidene derivatives of barbituric acids **50** with ethyl vinyl ether **51** were investigated at room temperature and pyrano[2,3-*d*]pyrimidines **52** and **53** were obtained in excellent yields (Scheme 9) [84]. Three-component one-pot syntheses of fused uracils were performed in aqueous suspensions. “On water” reactions of barbituric acids **50**, aldehydes **54**, and ethyl vinyl ether **51** where carried out at ambient temperature, whereas the one-pot synthesis with barbituric acids **50**, aldehydes **54**, and styrene or *N*-vinyl-2-oxazolidinone **56** required the heating of aqueous suspensions at 60 °C (Scheme 9). Formation of the unexpected side products **55** can be explained as the result of three-component reactions of barbituric acids and acetaldehyde which were produced from reaction of etyl vinyl ether and water, and ethyl vinyl ether **51**. Described “on water” cycloadditions were characterized by higher diastereoselectivity in contrast to reactions carried out in homogenous organic media (dichloromethane, toluene, Scheme 9). They allowed the *cis* adducts **57** to be obtained preferentially or exclusively. Green methods presented in this study avoid the use of catalysts, the heating of reaction mixtures for long time at high temperature, and the use of organic solvents.

#### Catalytic Hetero-Diels–Alder Reactions of 1-Oxa-1,3-Butadienes with Achiral Lewis Acids

**Scheme 10:**
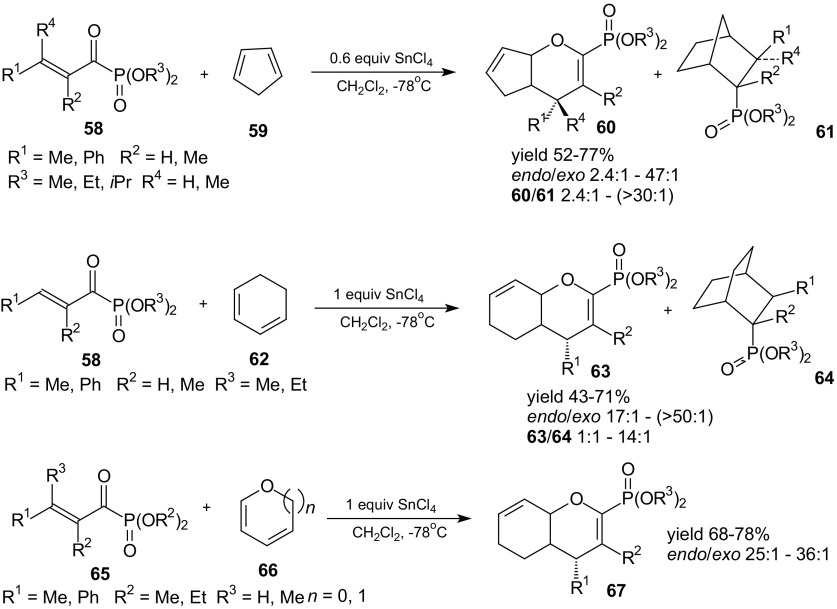
Lewis acid SnCl_4_ promoted HDA reactions of α-ketophosphonoenoates **58** and **65** with dienes **59**, **62** and **66**

It was mentioned in the Introduction that Lewis acids accelerate the HDA reactions of 1-oxa-1,3-butadienes [41–53]. Lewis acids can also improve regioselectivity and diastereoselectivity of these reactions. The example of catalytic HDA reaction are cycloadditions of α-keto-β,γ-unsaturated phosphonates **58** and **65** with cycloalkenes: cyclopentadiene **59**, cyclohexadiene **62**, dihydrofuran, and dihydropyran **66**, described by Hanessian and Compain (Scheme 10) [85]. The reactions led to the formation of the hetero-Diels–Alder products **60**, **63** and **67** in addition to the normally expected Diels–Alder cycloadducts **61** and **64**. Hetero-Diels–Alder cycloadducts with the *endo* product as the major isomer were the main products in the presence of SnCl_4_ as a Lewis acid. The effect of substituents on stereochemistry of these reactions can be explained by considering steric interactions in the transition state. Increasing the bulk of the ester moiety lowered the ratio of hetero to normal Diels–Alder products while geminal substitution favored the product formed by HDA reaction. In the reactions of dialkyl α-crotonylphosphonates **65** with dihydrofuran and dihydropyran **66**, the only isolable products were heterocycloadducts with high *endo*/*exo* ratios and good yields.

**Scheme 11:**
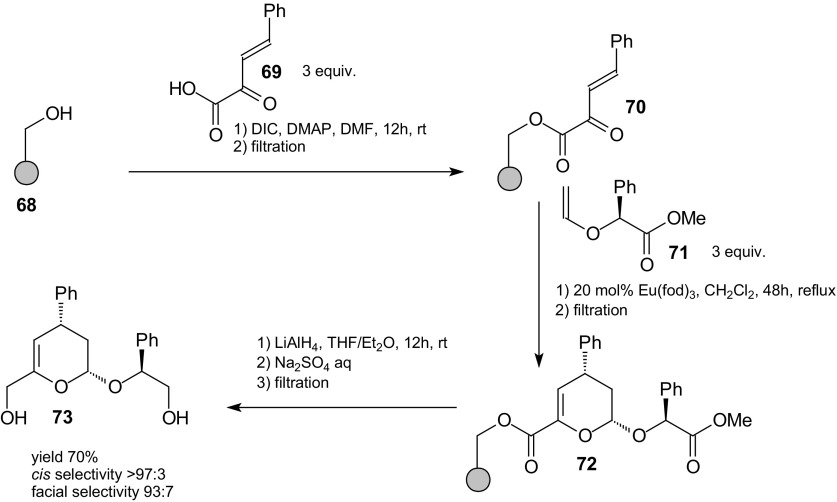
Eu(fod)_3_-catalyzed solid-phase HDA reaction of benzylidenepyruvate **70** with (*S*)-(+)-*O*-vinyl mandelate **71**

Compatibility of the carrier and the linker with the Lewis acid is a main criterion for a successful application of Lewis acid catalysts on solid supports in asymmetric [4 + 2] heterocycloadditions. Dujardin et al. demonstrated the usefulness of a Wang resin-bound heterodiene benzylidenepyruvate **70** for Eu(fod)_3_-catalyzed inverse-electron-demand HDA reactions with (*S*)-(+)-*O*-vinyl mandelate **71** (Scheme 11) [86]. The solid-phase sequence allowed an unprecedented reuse of the catalyst in the presence of excess dienophile in solution. Also, attempts with ethyl vinyl ether as an achiral dienophile gave positive results.

**Scheme 12:**
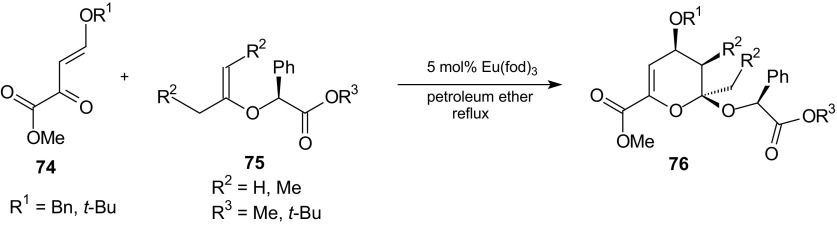
Eu(fod)_3_ catalyzed HDA reaction of alkylidenepyruvate **74** with *O*-isopropenyl mandelic esters **75**

Gong et al. examined asymmetric inverse-electron-demand HDA reaction of trisubstituted chiral enol ether **75** derived from (*R*)-mandelic acid (Scheme 12) [87]. Chiral 1,2,3,5-substituted tetrahydropyrans were synthesized by a three-step sequence with a remarkable and unprecedented *endo* and facial stereocontrol. The key step involved the Eu(fod)_3_-catalyzed HDA reaction of a trisubstituted chiral enol ether **75** and an activated heterodiene **74**. The stereoselective hydrogenation of the heteroadducts 1-alkoxydihydropyrans **76** was optimized by using Pd on charcoal and diisopropylethylamine, leading to a unique isomer [87].

**Scheme 13:**
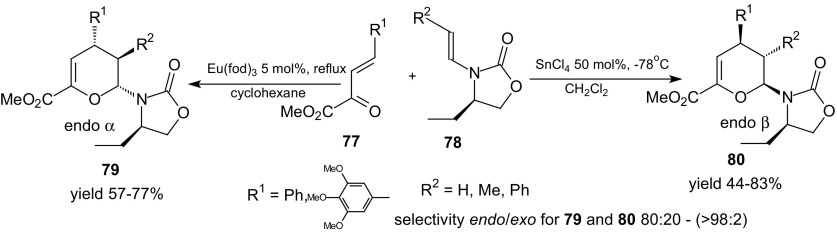
Lewis acid tuned facial stereodivergent HDA reactions of β-substituted *N*-vinyl-1,3-oxazolidin-2-ones **78**

Another example of inverse-electron-demand HDA reaction of 1-oxa-1,3-butadienes is the [4 + 2] acido-catalyzed heterocycloaddition between β-substituted *N*-vinyl-1,3-oxazolidin-2-ones **78** and unsaturated α-ketoesters **77** (Scheme 13) [88, 89]. Cycloadditions afforded dihydropyrans **79** and **80** with high levels of *endo* and facial selectivities.

A complete reversal of facial differentiation was achieved by using a different Lewis acid, leading to the stereoselective formation of either *endo*-α **79** or *endo*-β **80** adducts. The *endo*-α adduct **79** was obtained with using Eu(fod)_3_ as the catalyst and *endo*-β adducts **80** was the main product if the promoter was SnCl_4_ (Scheme 13) [88, 89].

#### Enantioselective Approach: Catalytic Enantioselective Hetero-Diels–Alder Reactions of 1-Oxa-1,3-Butadienes with Chiral Lewis Acids

**Scheme 14:**
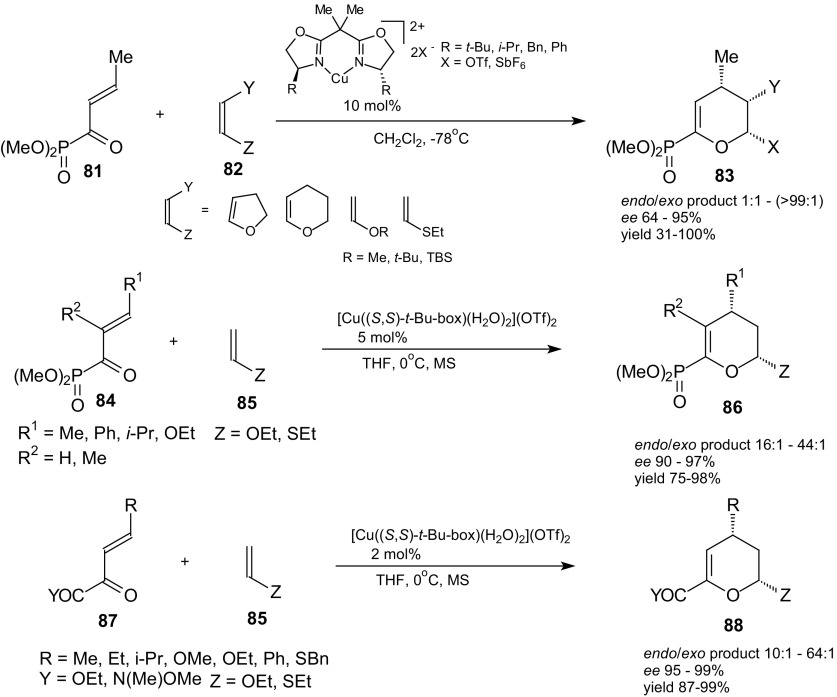
HDA reactions of acyl phosphonates **81**, **84** and *β*,*γ*-unsaturated *α*-keto esters  and amides **87** with enol ethers and sulfides **82** and **85** catalyzed by bis(oxazoline)-Cu(II) complexes

The catalytic enantioselective HDA reactions of 1-oxa-1,3-butadienes with chiral Lewis acids were widely explored reactions. The chiral bisoxazoline copper(II) complexes have been shown to be effective catalysts for inverse-electron-demand HDA reactions. The reactions of α,β-unsaturated acyl phosphonates **81** and **84** and β,γ-unsaturated α-keto esters and amides **87** with enol ethers and sulfides **82** and **85** as dienophiles were described by Evans et al. (Scheme 14) [90]. The products were prepared with high diastereo- and enantioselectivity. The selectivities of reactions exceeded 90 % even at room temperature. The synthesis of bicyclic adducts **83** in high diasteromeric and enantiomeric excess proved that cyclic enol ethers **82** can be excellent dienophiles. The derived cycloadducts were transformed to useful chiral building blocks such as desymmetrized glutaric acid derivatives or highly functionalized tetrahydropyran products. The authors examined that the high diastereoselectivity for catalyzed HDA reactions is a result of the *endo* orientation of dienophiles.

**Scheme 15:**
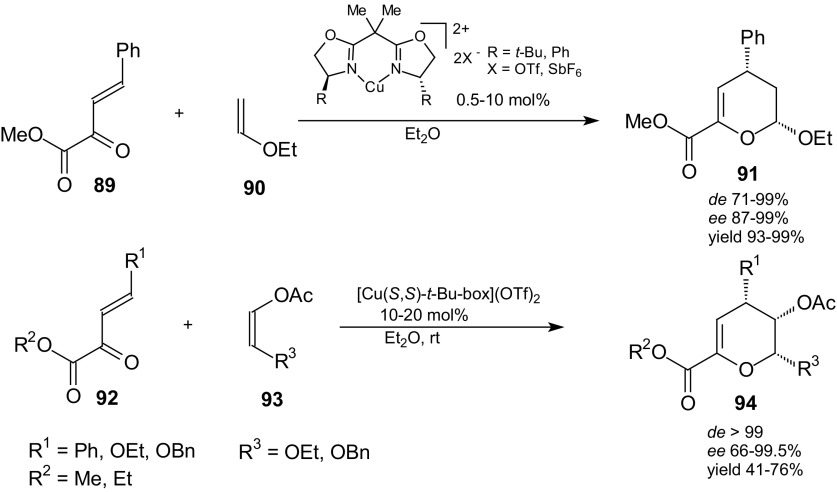
HDA reactions of γ-substituted β,γ-unsaturated α-keto esters **89** and **92** with vinyl ether **90** and *cis*-disubstituted alkenes **93** catalyzed by bis(oxazoline)-Cu(II) complexes

A highly enantioselective approach for the synthesis of optically active carbohydrate derivatives by inverse-electron-demand HDA reaction of α,β-unsaturated carbonyl compounds with electron-rich alkenes catalyzed by combination of chiral bisoxazolines and Cu(OTf)_2_ as the Lewis acid was also presented by Jorgensen et al. [91]. The reaction of unsaturated α-keto esters **89** and **92** with vinyl ether **90** and various types of *cis*-disubstituted alkenes **93** proceeded in good yield, high diastereoselectivity, and excellent enantioselectivity (Scheme 15). The potential of the reaction was demonstrated by the synthesis of optically active carbohydrates such as spiro-carbohydrates, an ethyl β-d-mannoside tetraacetate, and acetal-protected *C*-2-branched carbohydrates [91].

**Scheme 16:**
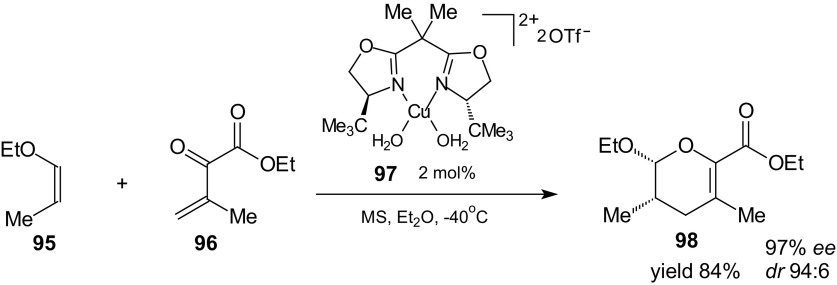
Enantioselective HDA approach to the synthon of azaspiracid

Catalytic enantioselective HDA reaction of 1-oxa-1,3-butadiene with inverse-electron-demand was used in synthesis of the marine neurotoxin-(+)-azaspiracid [92]. Cycloaddition between two components of the HDA reaction **95** and **96** proceeded readily using 2 mol% loadings of the hydrated copper complex **97** (Scheme 16). Catalyst **97** was dehydrated with molecular sieves prior to use. Diethyl ether was the optimal solvent for this HDA reaction (97 % *ee*, *dr* 94:6). The desired cycloadduct **98** was isolated in 84 % yield as a single isomer.

**Scheme 17:**
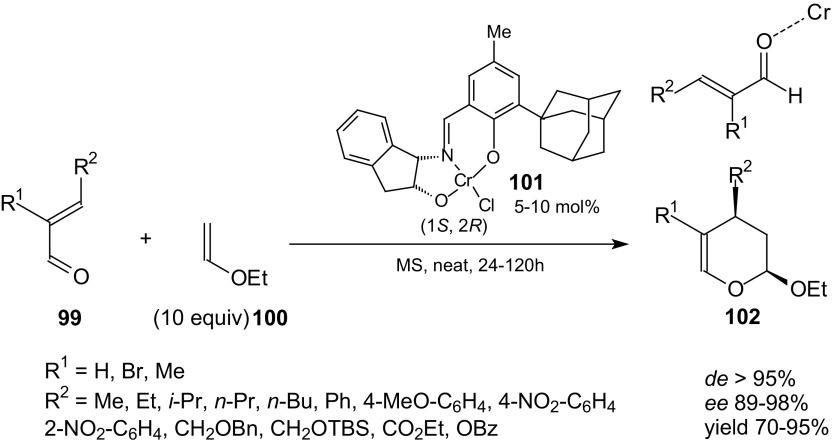
HDA reactions catalyzed by (Schiff base)Cr(III) catalyst **101**. Model of one-point binding of an α,β-unsaturated aldehydes **99** to a Lewis acid center

The tridentate (Schiff base) chromium complex has been identified as a highly diastereoselective and enantioselective catalyst in HDA reactions between aldehydes and mono-oxygenated 1,3-diene derivatives [93]. Jacobsen et al. examined if use of this chiral catalyst can be evaluated for the reactions of conjugated aldehydes [94]. The inverse-electron-demand HDA reactions of crotonaldehyde and the wide range of α,β-unsaturated aldehydes **99** bearing β substituents and vinyl ether **100** proceeded in the presence of molecular sieves MS with 5 mol% chiral catalyst **101** at room temperature to provide cycloadducts **102** with excellent diastereoselectivity, enantioselectivity, and in high yield (Scheme 17).

A dramatic improvement was observed in reactions carried out under solvent-free conditions and excess ethyl vinyl ether **100**. Usage of solvents generally resulted in significantly lower enantioselectivity in the cycloaddition. As the steric bulk of the alkyl group of dienophile was increased, the selectivity and reactivity decreased. The optimal dienophile was ethyl vinyl ether. In the solid state, catalyst **101** exists as a dimeric structure, bridged through a single water molecule and bearing one terminal water ligand on each chromium center. Opening of a coordination site by dissociation of the terminal water molecule for complexation of the aldehyde substrate explains the important role of molecular sieves in these reactions [95, 96].

**Scheme 18:**
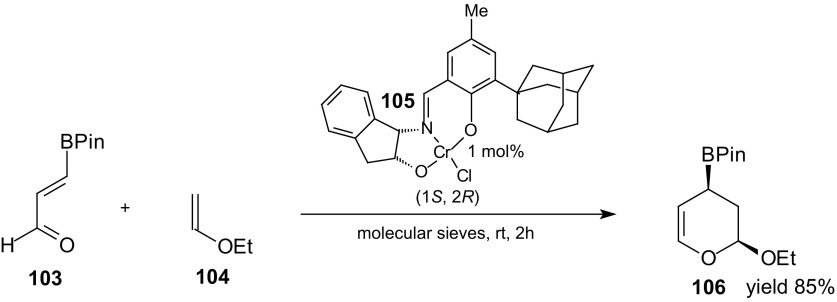
Asymmetric inverse-electron-demand HDA reaction of 3-boronoacrolein pinacolate **103** and ethyl vinyl ether **104** as a key step in synthesis of several members of the styryllactone family

Asymmetric inverse-electron-demand HDA reaction of 1-oxa-1,3-butadienes was a key step in synthesis of several members of the bioactive styryllactone family [97]. Treatment of ethyl vinyl ether **104** with 3-boronoacrolein pinacolate **103** in the presence of Jacobsen’s [(Schiff base)chromium(III)] complex **105** resulted in the formation of cycloadduct **106** in good yield (85 %) and high enantioselectivity (96 % *ee*; Scheme 18). This strategy can be applicable to the synthesis of different stereoisomers by taking into account both isomers of mandelic acid and the different chromium(III) complexes.

#### Enantioselective Approach: Catalytic Hetero-Diels–Alder Reactions of 1-Oxa-1,3-Butadienes with Chiral Organocatalysts

**Scheme 19:**
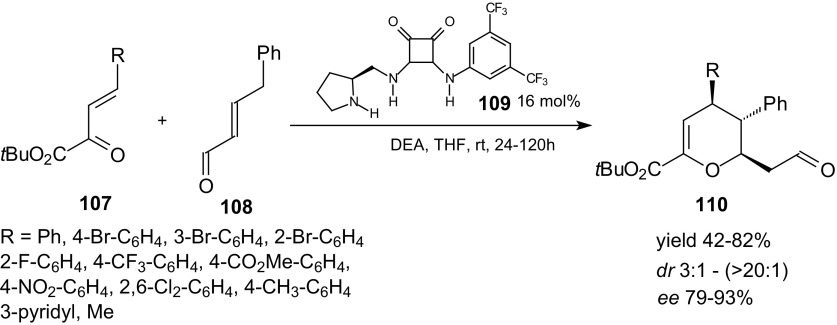
HDA reactions between β,γ-unsaturated α-ketoesters **107** and (*E*)-4-phenylbut-2-enal **108** in the presence of aminocatalyst

In recent years, organocatalysis has been established as a very powerful tool for the synthesis of functional molecules. Asymmetric versions of HDA utilizing chiral organocatalysts have been developed. Asymmetric aminocatalytic strategies involving HOMO activation of the dienophile constitute an important alternative to classical LUMO-lowering pathways [98–100]. Albrech et al. developed the first H-bond-directed inverse-electron-demand HDA proceeding via a dienamine intermediate [101, 102]. They evaluated the organocatalytic reaction between various β,γ-unsaturated α-ketoesters **107** and (*E*)-4-phenylbut-2-enal **108** in the presence of various aminocatalysts (Scheme 19). The H-bond-directing dienamine catalyst **109** promoted the inverse-electron-demand HDA reactions.

In most of the cycloadditions of **107** and **108**, good yields and high regio- and stereoselectivities were obtained. High stereoselectivities were observed by employing a bifunctional squaramide-containing aminocatalyst **109**. The authors postulated that dienamine intermediate is formed by condensation of aminocatalyst **109** with the α,β-unsaturated aldehyde **108**, and the next heterodiene **107** in *s*-*trans* conformation is recognized by the catalyst. Two cycloreactants **107** and **108** are activated through H-bond interactions and are positioned to facilitate the cycloaddition step.

**Scheme 20:**
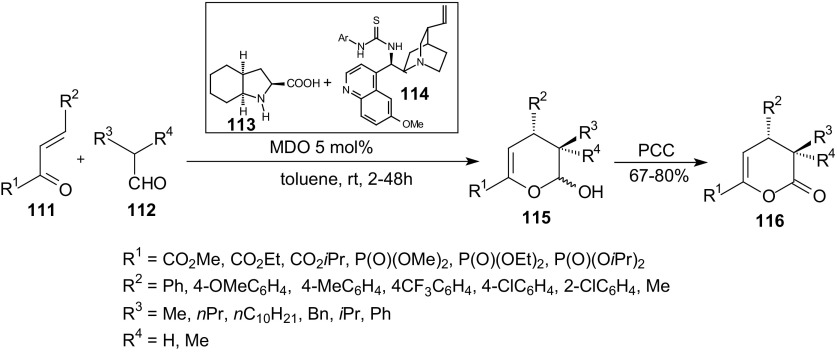
MDO catalyzed HDA reactions of electron-deficient enones **111** and aldehydes **112**

Most recently, there was considerable interest in applying self-assembled organocatalysts/modularly designed organocatalysts (MDO) in catalytic reactions. Zhao et al. demonstrated that MDO self-assembled from proline derivatives and cinchona alkaloid derived thioureas are highly efficient catalysts for inverse-electron-demand HDA reactions [103]. They developed highly enantioselective HDA reactions of electron-deficient enones **111** and aldehydes **112** by using MDO (Scheme 20). Quinidine and (2*S*, 3a*S*, 7a*S*)-octahydro-1*H*-indole-2-carboxylic acid (OHIC) are both poor catalysts for the inverse-electron-demand HDA reactions between aldehydes and electron-deficient enones. However, forming MDO by their self-assembly with cinchona alkaloid-derived thioureas can improve the efficiency, reactivity and stereoselectivity of these catalysts. Various aldehydes **112**, including long-chain and branched aldehydes, were found to be excellent substrates for the MDO-catalyzed HDA reactions (Scheme 20).

The high yield and enantioselectivity of the reactions was restored (up to 95 % yield and 95 % *ee*). The ester alkyl group of β,γ-unsaturated α-ketoesters **111** has almost no influence on either the reactivity or enantioselectivity. Similarly, the substituent on the phenyl ring of the enones **111** has minimal effects on the reactivity and the asymmetric induction of these reactions. β,γ-Unsaturated α-ketophosphonates **111** may also be applied in these reactions if a higher loading of the precatalyst modules (10 mol%) is used. The authors proposed a plausible transition state on the basis of the product **116** stereochemistry and the MDO structure [103]. They showed that the aldehyde **112** reacts with the OHIC moiety of the MDO to form an (*E)*-enamine. Next, the thiourea moiety of the MDO forms hydrogen bonds with the enone **111** and directs to enamine from the front. The attack of the enone **111** onto the *Re* face of the enamine in an *endo* transition state leads to the formation of the observed (4*S*, 5*R*)-product **116**.

#### Enantioselective Approach: Hetero-Diels–Alder Reactions of 1-Oxa-1,3-Butadienes with Chiral Auxiliaries

**Scheme 21:**
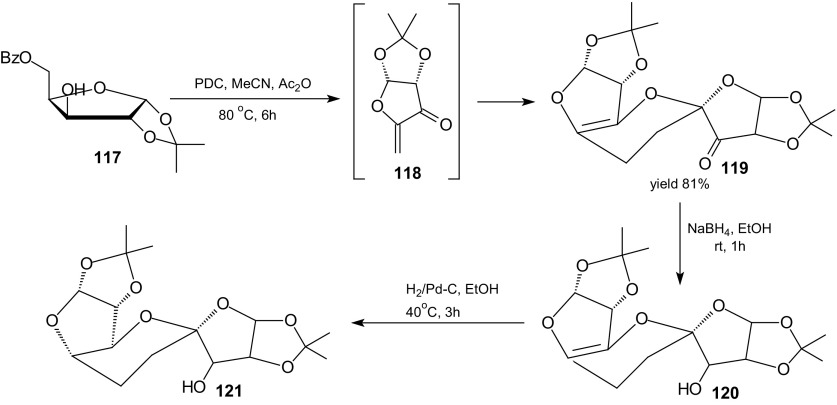
Stereoselective synthesis of higher carbon sugar **121** from protected D-xylose **117** by HDA reaction of the α,β-unsaturated ketone **118**

Inverse-electron-demand HDA reaction between 1-oxa-1,3-butadienes and electron-rich alkenes represents one of the most direct approaches for the synthesis of optically active carbohydrate derivatives. To obtain optically active dihydropyrans derivatives by the HDA approach, either a catalytic enantioselective reaction or a chiral transformation via the use of a chiral auxiliary is necessary. The enantioselective HDA reaction requires chiral 1-oxa-1,3-butadienes or optically active alkene. The HDA reaction of the α,β-unsaturated ketone **118** prepared in situ from protected D-xylose **117** was used as the key step for the synthesis of a C10 higher carbon sugar **119** in a one-pot multi-step route (Scheme 21) [104].

Two molecules of α,β-unsaturated ketone **118** undergo the HDA reaction affording the 10 carbon sugar **119**. Reduction and catalytic hydrogenation of cycloadduct **119** gave stereoselectively a single product **121** in an excellent yield.

**Scheme 22:**
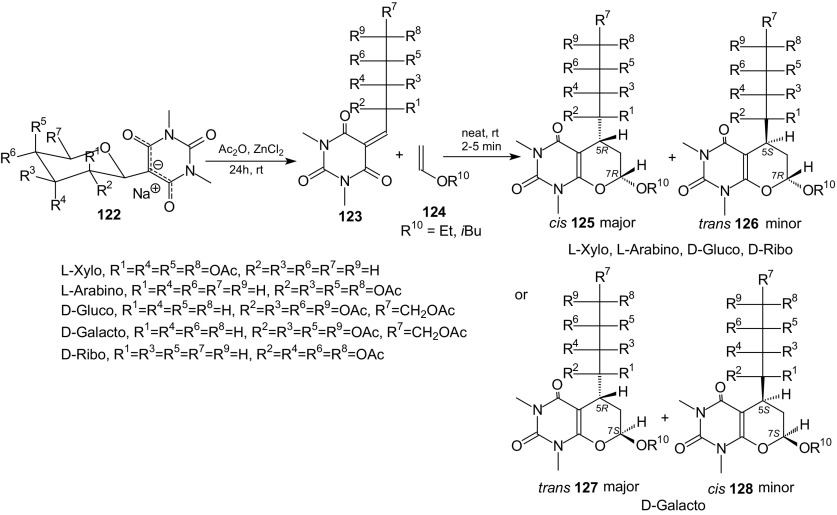
Acetylation of *C*-glycosides **122** and HDA reactions of 5-ylidene derivatives **123** with enol ethers **124**. Synthesis of pyrano[2,3-*d*]pyrimidines **125**-**128** with a sugar moiety

**Scheme 23:**
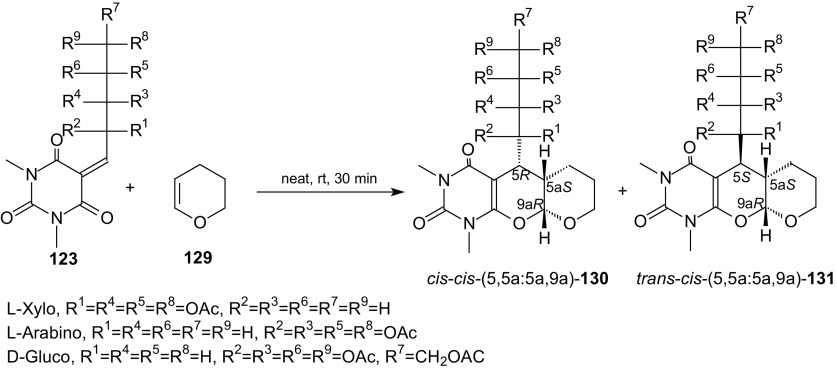
HDA reactions of *O*-acetylated 1,3-dimethyl-2,4,6-trioxo-pyrimidin-5-ylidene alditols **123** with cyclic enol ether **129**

Recently, it was shown that fused uracils, such as pyrano[2,3-*d*]pyrimidines with an aryl substituent at carbon C(5) in the ring system can be efficiently synthesized by HDA reactions of 5-arylidene derivatives of barbituric acids with vinyl ethers [82]. To increase the potential pharmacological activity of the fused uracil, a sugar moiety can be introduced instead of an aryl group at the C(5) position of pyrano[2,3-*d*]pyrimidine. Therefore, 5-ylidene barbituric acids bearing the carbohydrate substituent were constructed. A convenient and efficient procedure for the preparation of fused uracils containing a sugar moiety was described [105]. The reaction sequence was: Knoevenagel condensation of unprotected sugars and barbituric acid in water, acetylation of *C*-glycosides and HDA reaction. The cycloaddition reactions of *O*-acetylated 1,3-dimethyl-2,4,6-trioxo-pyrimidin-5-ylidene alditols **123**, representing an 1-oxa-1,3-butadiene system, with enol ethers **124** were performed in the absence of solvent at room temperature for 2–5 min and enantiomerically pure *cis* and *trans* diastereoisomers of pyrano[2,3-*d*]pyrimidines **125**–**128** with an alditol moiety were obtained in good 80–87 % yields (Scheme 22). It is worth noting that barbituric acid 5-ylidene alditols **123** are extremely reactive because they underwent smooth HDA reactions at a temperature of −80 °C as well as at room temperature. Observed diastereoselectivity of the HDA reactions of **123** and **124** changed in the range from 6.6:1 to 2.1:1. Cycloadducts *cis* were the major products in all reactions except those of D-galactose derivatives (Scheme 22). The inverse-electron-demand HDA reactions of *O*-acetylated 5-ylidene derivatives **123** with a tenfold excess of cyclic enol ether **129** were performed in solvent-less conditions at room temperature for 30 min, and pyrano[3′,2′:5,6]pyrano[2,3-*d*]pyrimidines **130** and **131** were obtained in good 76–78 % yields (Scheme 23). *O*-Acetylated 1,3-dimethyl-2,4,6-trioxo-pyrimidin-5-ylidene alditols **123** can act as active heterodienes in HDA reactions and their use in cycloadditions allows preparation of the enantiomerically pure diastereoisomers of pyrano[2,3-*d*]pyrimidines with a sugar moiety.

**Scheme 24:**
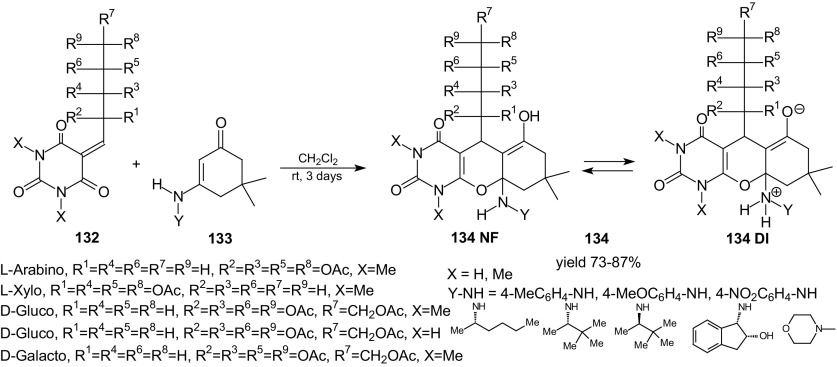
HDA reactions of alditols **132** with dimedone enamines **133**. Synthesis of fused uracils - chromeno[2,3-*d*]pyrimidine-2,4-diones **134**

In the field of pericyclic reactions, the development of new cycloreactants is a continuous challenge. Dimedone enamines were applied as new dienophiles in HDA reactions with inverse-electron-demand of 1-oxa-1,3-butadienes [106]. Cycloadditions of barbituric acid 5-ylidene alditols **132**, representing a 1-oxa-1,3-butadiene system, with dimedone enamines **133** were performed in dichloromethane at room temperature for 3 days, and fused uracils–chromeno[2,3-*d*]pyrimidine-2,4-diones **134** were prepared in good (73–87 %) yields (Scheme 24). Only one enantiomerically pure stereoisomer was obtained in each studied cycloaddition. Analysis of proton nuclear magnetic resonance ( ^1^H NMR) and two-dimensional (2D) NMR spectra allowed for the determination that cycloadducts **134** exist in solution as a mixture of the neutral form **134 NF** and dipolar ion **134 DI**. The prepared fused uracils, possessing both amine and enol functional groups, share amphiprotic properties and are zwitterions in solid state. Important for biological interaction, groups such as different sugar moieties, enol moieties and different amino groups can be introduced into fused uracil systems by this simple HDA reaction. It was also shown that different alkenes can be used as dienophiles towards barbituric acid 5-ylidene alditols **132**; for example, styrene or 1-amino-2-thiocarbamoyl-cyclopent-1-ene [106].

**Scheme 25:**
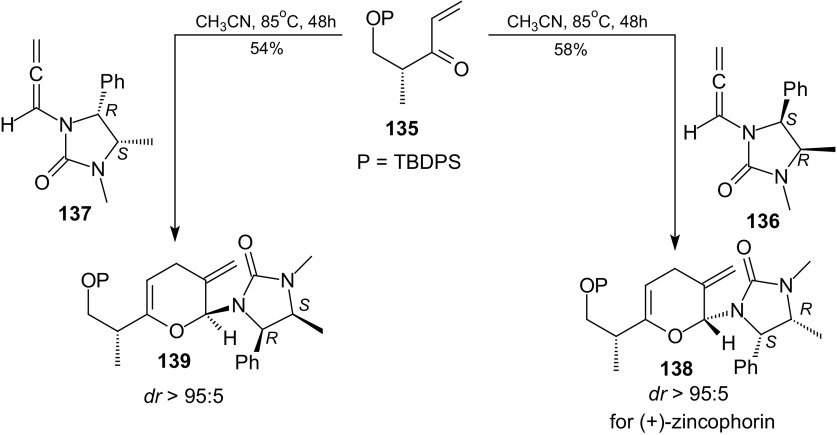
HDA reactions of chiral enone **135** with chiral allenamides **136** and **137**. The key step in synthesis of the C1-C9 subunit of (+)-zincophorin

The application of stereoselective inverse-electron-demand HDA reaction of 1-oxa-1,3-dienes and chiral allenamides in natural product synthesis was described by Song et al. [107]. They used this reaction as a key step in synthesis of the C1–C9 subunit of (+)-zincophorin (Scheme 25).

Both reactions **136** with **135** and **137** with **135** provided respectively pyrans **138** and **139** in 58 and 54 % yields, as single isomers, after heating in a sealed tube at 85 °C for 48 h in acetonitrile as the solvent (Scheme 25).

### Intramolecular Hetero-Diels–Alder Reactions of 1-Oxa-1,3-Butadienes

#### Two-Component Domino Knoevenagel Hetero-Diels–Alder Reactions of 1-Oxa-1,3-Butadienes with an Intramolecular Cycloaddition

**Scheme 26:**
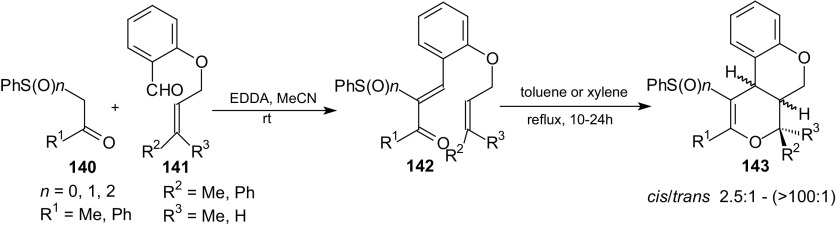
Domino Knoevenagel intramolecular HDA reactions of sulfenyl, sulfinyl and sulfonyl activated methylene compounds **140** with 2-alkenyloxy aromatic aldehydes **141**. Synthesis of polycyclic 2*H*-pyran derivatives **143**

The domino Knoevenagel intramolecular hetero-Diels–Alder reaction is one of the most powerful synthetic routes for the synthesis of various heterocycles and natural products. This reaction can be used in dihydropyran synthesis [54–68]. In intramolecular cycloaddition, the 1-oxa-1,3-butadienes are prepared in situ by Knoevenagel condensation of aldehydes possessing the dienophile moiety and a 1,3-dicarbonyl compound. A lot of different aldehydes and 1,3-dicarbonyl compounds such as barbituric acids, Meldrum’s acid, 1,3-cyclohexanedione, dimedone, 4-hydroxycoumarin, indiandiones, pyrazolones, or isooxazolones can be used. *Cis*-fused cycloadducts are the main products in intramolecular HDA reactions of oxabutadienes obtained from aromatic aldehydes [54, 55]. Reactions of oxabutadienes derived from aliphatic aldehydes result in the *trans*-fused cycloadducts [56, 57]. In recent years, intramolecular HDA reactions of 1-oxa-1,3-butadienes have been used widely in numerous reactions in organic synthesis due to their economical and stereo-controlled nature. These reactions allow the formation of two or more rings at once, avoiding sequential chemical transformations. Therefore, the scope of the intramolecular HDA reactions of 1-oxadienes was expanded recently. The influence of an electron-withdrawing group at C-3 in 1-oxa-1,3-butadienes on the intramolecular HDA reaction was studied. First, the influence of cyano, carbonyl, and ethoxycarbonyl groups was examined [108]. Next, it was demonstrated that sulfur-containing substituents incorporated into 1-oxa-1,3-butadienes positively influence the results of the cycloaddition. The intramolecular HDA reactions of sulfenyl-, sulfinyl-, and sulfonyl-activated methylene compounds **140** with 2-alkenyloxy aromatic aldehydes **141** were conducted (Scheme 26) [109].

Knoevenagel condensations of 1-(phenylsulfenyl)-, 1-(phenylsulfinyl)-, and 1-(phenylsulfonyl)-2-propanones **140** with 2-alkenyloxy aromatic aldehydes **141** yielded the corresponding condensation products **142** which in turn underwent intramolecular HDA reactions during heating in boiling toluene or xylene (Scheme 26). *Cis*-fused 2*H*-pyran derivatives **143** were the major products. An increase of the reactivity and a decrease of the diastereoselectivity of the HDA reactions were observed in order: PhS derivative, PhSO_2_ derivative and compounds containing PhSO group [108, 109].

**Scheme 27:**
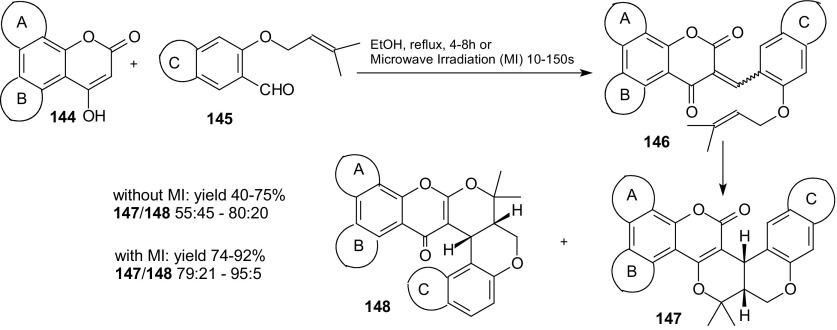
Microwave accelerated domino Knoevenagel intramolecular HDA reactions of 4-hydroxy coumarin and its benzo-analogues **144** with *O*-prenylated aromatic aldehydes **145**

The most widely used 1-oxa-1,3-butadienes in intramolecular HDA reactions are usually those where the double bond is placed between the symmetrical 1,3-dicarbonyl compounds. Shanmugasundaram et al. studied the heterocycloaddition in which the alkene part was flanked by a keto carbonyl and a lactone carbonyl [110]. The reactions of 4-hydroxy coumarin and its benzo-analogues **144** with *O*-prenylated aromatic aldehydes **145** were examined (Scheme 27). Pyrano fused polycyclic compounds **147** and **148** were prepared with a high degree of chemoselectivity by the application of microwave irradiation. These reactions offers an easy access to pyrano[3,2-*c*]coumarin **147** which is a structural element of many natural products.

Chemoselectivity was achieved with the reduction in reaction time because the cycloadducts **147** and **148** formed in the ratios ranging from 79:21–95:5 when the reactions were carried out under microwave irradiation for 10–150 s. Reactions of unsymmetrical 1,3-diones **144** with citronellal were also described [110].

**Scheme 28:**
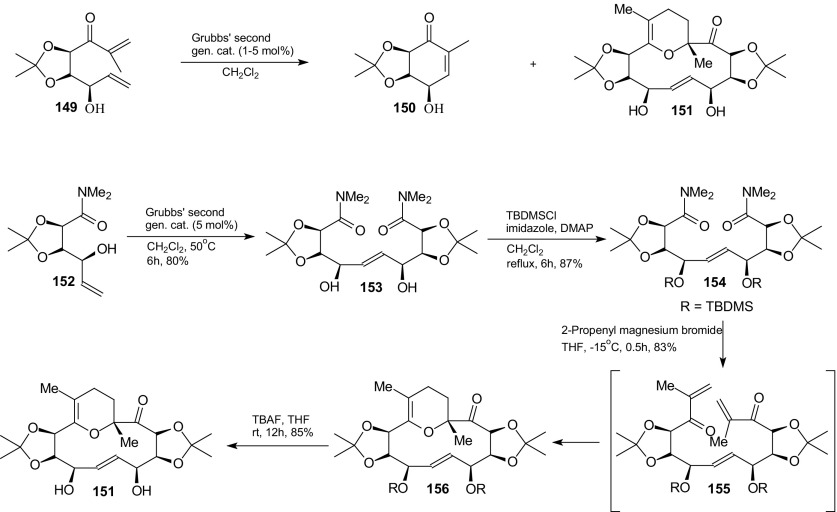
Synthesis of macrocycle **151** by sequential cross metathesis and HDA reaction

Surprising formation of a 2,3-dihydro-4*H*-pyran containing 14-membered macrocycle **151** by sequential olefin cross metathesis and a highly regiospecific intramolecular HDA reaction of 1-oxa-1,3-dienes was described by Prasad and Kumar (Scheme 28) [111]. They studied the reaction of a hydroxydienone **149** derived from tartaric acid with Grubbs’ second generation catalyst. Presence of the unprotected hydroxyl group in the hydroxyenone led to the formation of macrocycle **151**. Protection of the hydroxyl group resulted in the ring-closing metathesis product **150**.

The authors made the experiment to show that obtaining macrocycle **151** involves the formation of intermediate **155**. Dimerization of hydroxyamide **152** by olefin cross metathesis with Grubbs second generation catalyst gave the bis-amide **153** (Scheme 28). Protection of the two hydroxyl groups in **153** as the bis-silyl ether **154** and then the reaction with 2-propenylmagnesium bromide resulted in formation of the macrocycle **156**. Deprotection of the silyl ethers in **156** furnished the macrocycle **151** in 85 % yield. These studies represent the first example of a tandem olefin cross metathesis HDA reaction sequence.

**Scheme 29:**
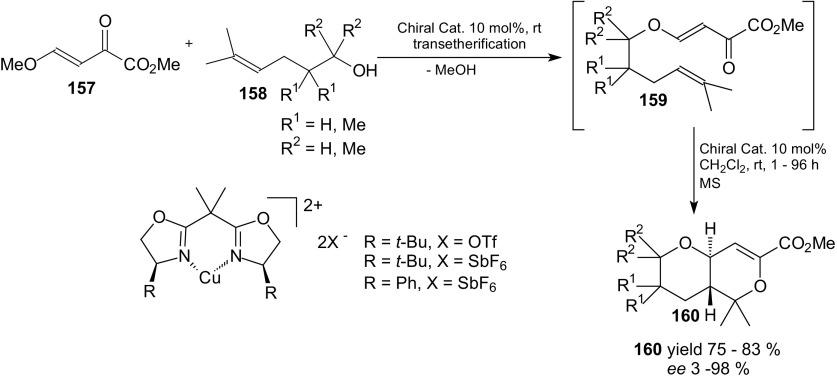
Catalylic enantioselective tandem transetherification-intramolecular HDA reaction of methyl (*E*)-4-methoxy-2-oxo-3-butenoate **157** with δ,ε-unsaturated alcohols **158**

Wada et al. developed a new type of intramolecular HDA reaction of 1-oxa-1,3-butadienes–tandem transetherification-intramolecular HDA reaction. Heterodienes were obtained in situ by a transetherification under thermal conditions from β-alkoxy-substituted α,β-unsaturated carbonyl compounds bearing an electron-withdrawing substituent and δ,ε-unsaturated alcohols [112]. This tandem reaction proceeded stereoselectively to afford *trans*-fused hydropyranopyrans. Next, chiral Lewis acid catalysts were used in this new type of transformation. Wada et al. examined the catalytic asymmetric tandem transetherification-intramolecular HDA reaction of methyl (*E*)-4-methoxy-2-oxo-3-butenoate **157** with δ,ε-unsaturated alcohols **158** (Scheme 29) [113]. The optically active catalyst derived from the (*S*,*S*)-*tert*-Bu-bis(oxazoline) and Cu(SbF_6_)_2_ in presence of molecular sieves was a highly effective Lewis acid catalyst. The *trans*-fused hydropyranopyran derivatives **160** were prepared in yields up to 83 % and with high enantiomeric excess up to 98 %. In order to prevent the acid-induced cyclization, molecular sieves were used as a dehydratation agent.

**Scheme 30:**
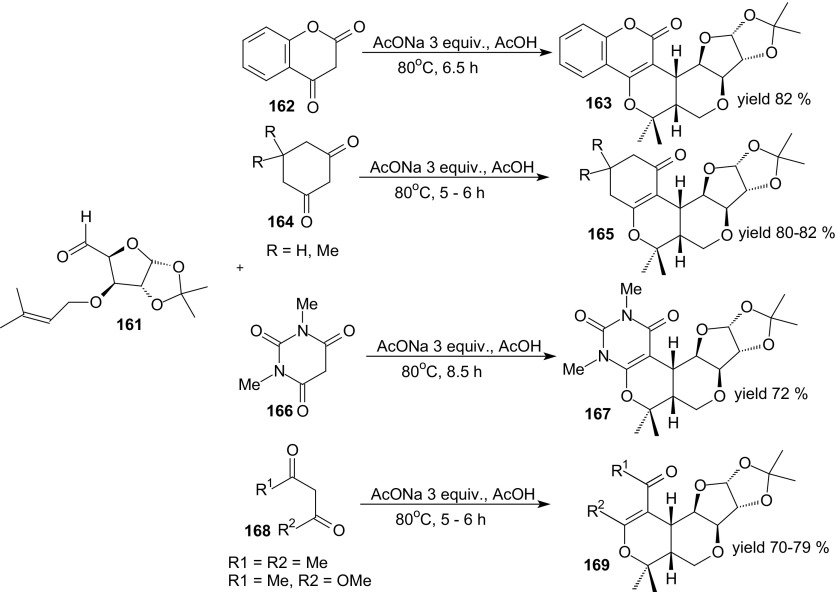
Domino Knoevenagel HDA reactions of sugar aldehyde **161** with 4-hydroxycoumarin **162**, cyclohexa-1,3-dione or dimedone **164**, 1,3-dimethylbarbituric acid **166** and 1,3-diones **168**

Yadav et al. presented the synthesis of carbohydrate analogues, *cis*-fused chiral polyoxygenated (tricyclic, tetracyclic, and pentacyclic) heterocycles by domino Knoevenagel intramolecular HDA reactions [114]. The *O*-prenyl derivative of a sugar aldehyde **161** derived from d-glucose underwent reactions with 1,3-diones **162**, **164**, **166** and **168** in presence of sodium acetate in acetic acid at 80 °C (Scheme 30). The reactions were highly stereoselective affording exclusively *cis*-fused furopyranopyrans **163**, **165**, **167** and **169** in 70–82 % yields. The authors suggested that the cycloadditions proceeded in a concerted manner via an *endo*-*E*-*syn* transition state.

#### Catalytic Intramolecular HDA Reaction of 1-Oxa-1,3-Butadienes and Alkynes

**Scheme 31:**
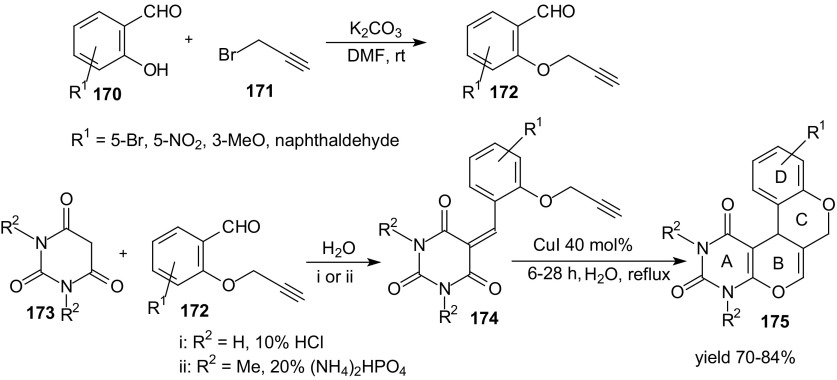
Synthesis of *O*-propargylated aldehydes **172** and fused uracils - oxa-helicene derivatives **175** by intramolecular HDA reaction

Due to the lower reactivity of alkynes in comparison to the corresponding alkenes, no HDA reaction of 1-oxa-1,3-butadienes with alkynes has been reported. Recently, different Lewis acids have provided new opportunities for various catalytic alkyne reactions. Some of the most frequently used transition metal catalysts are copper(I) compounds. Khoshkholgh et al. studied the intramolecular HDA reaction of 1-oxa-1,3-butadiene and an alkyne in the presence of CuI [115]. The Williamson reaction of propargyl bromide **171** and salicylaldehydes **170** afforded compounds **172** (Scheme 31). The 1-oxa-1,3-butadienes **174** were prepared through Knoevenagel reaction of *O*-propargylated salicylaldehyde derivatives **172** and barbituric acids **173** with yields between 75 and 94 %. Intramolecular HDA reactions were carried out in the presence of CuI (40 mol%) and water as the solvent and tetracyclic uracils **175** were prepared in 70–84 % yields. The authors explained that the initial step of cycloaddition was probably the formation of a π-complex with CuI since copper(I) salts can act as a π-electrophilic Lewis acid. This complexation of alkyne increases activity toward an 1-oxa-1,3-butadiene system (Scheme 31).

**Scheme 32:**
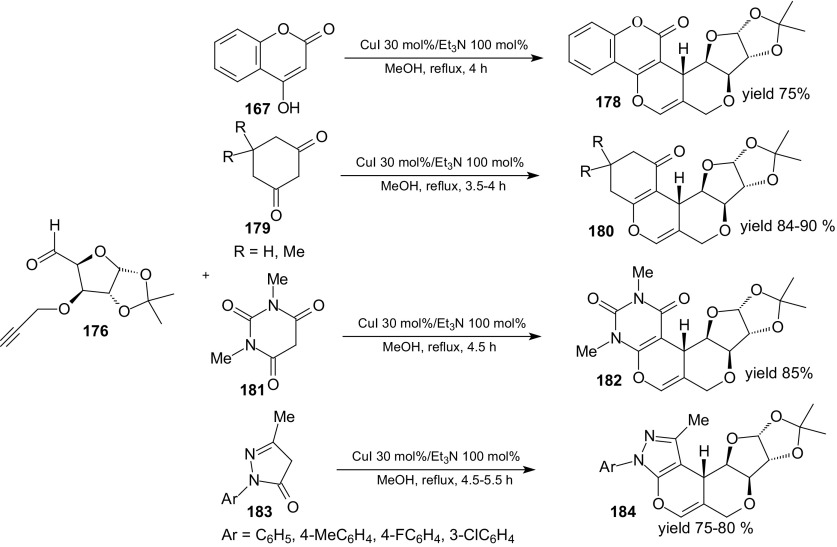
Domino Knoevenagel HDA reactions of sugar aldehyde **176** with 4-hydroxycoumarin **167**, cyclohexa-1,3-dione or dimedone **179**, 1,3-dimethylbarbituric acid **181** and 1-aryl-pyrazol-5-ones **183**

Yadav et al. developed a novel method for the synthesis of sugar-annulated tetracyclic- and pentacyclic-heterocycles in a single-step operation [116]. The *O*-propargyl derivative of a sugar aldehyde **176** derived from d-glucose undergoes smooth domino Knoevenagel intramolecular HDA reactions with 1,3-diketones **177**, **179**, **181** and 1-aryl-pyrazol-5-ones **183** in the presence of CuI/Et_3_N in refluxing methanol (Scheme 32).

Furopyranopyrans **178**, **180**, **182** and **184** were prepared in 75–90 % yields. The authors assumed that these cycloadditions proceed in a concerted way via an *endo*-*E*-*syn* transition state [116]. Acyclic 1,3-diketones such as acetyl acetone and ethyl acetoacetate can’t be used in the reaction.

**Scheme 33:**
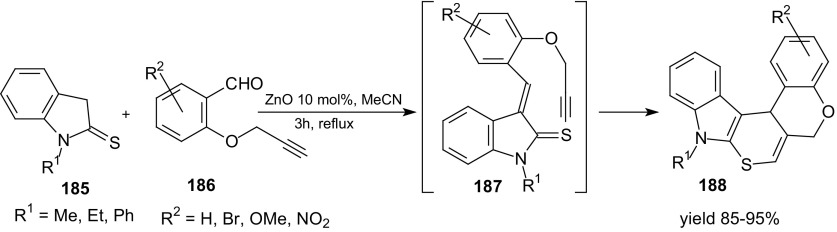
ZnO-catalyzed synthesis of indole-thiopyrano-chromene derivatives **188** via domino Knoevenagel intramolecular HDA reactions

Another example of catalytic intramolecular HDA reaction of 1-oxa-1,3-butadienes and an alkyne is domino Knoevenagel intramolecular HDA reaction of indolin-2-thiones **185** and *O*-propargylated salicylaldehyde derivatives **186** in the presence of ZnO (Scheme 33) [117].

The major advantage of this reaction is the fact that pentacyclic indole derivatives **188** can be isolated by filtration from the reaction mixture. This method also has advantages such as the use of commercially available, non-toxic and inexpensive ZnO as catalyst, low loading of catalyst, and high yields of products.

**Scheme 34:**
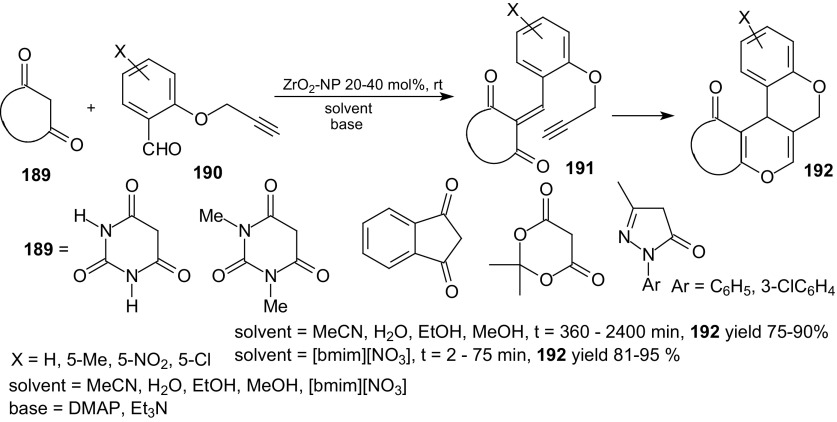
Domino Knoevenagel­ HDA reactions of *O*-propargylated salicylaldehydes **190** with active methylene compounds **189** in the presence of ZrO_2_-nanopowder

Balalaie et al. described domino Knoevenagel intramolecular HDA reactions of *O*-propargylated salicylaldehyde **190** with active methylene compounds **189** in the presence of ZrO_2_-nanopowder (NP) as a Lewis acid in ionic liquid and different organic solvents (Scheme 34) [118]. The reactions were carried out for different active methylene compounds **189** such as barbituric acid, *N,N*-dimethyl barbituric acid, indandione, Meldrum’s acid, and pyrazolone.

The solutions of aldehyde **190** and appropriate 1,3-dicarbonyl compound **189**, ZrO_2_ and base in ionic liquid or organic solvent were stirred for 5–40 min at room temperature and desired products were obtained in 80–95 % yields. The best results were obtained for 5-nitro-*O*-propargylated salicylaldehyde and 1-butyl-3-methylimidazolium nitrate [bmim][NO_3_] as the reaction medium. Balalaie et al. also used ionic liquid [bmim][NO_3_] in the presence of 30 mol% CuI in the domino Knoevenagel HDA reactions of *O*-propargylated salicylaldehydes with some active methylene compounds [119].

#### Two-Component Domino Knoevenagel Hetero-Diels–Alder Reactions of 1-Oxa-1,3-Butadienes with Intramolecular Cycloaddition in Water or Solvent-Free

**Scheme 35:**
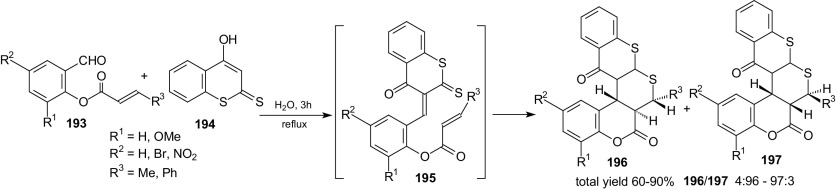
Synthesis thiochromone-annulated thiopyranocoumarins **196** and **197** by domino Knoevenagel HDA reaction

Water is the solvent of choice for nature to carry out syntheses of complex organic molecules. Water is a clean, inexpensive, environment friendly reaction medium. Therefore, the choice of water as the solvent for organic reactions in the laboratory synthesis is obvious. However, water as a solvent was ruled out from organic reactions. It has been changed in 1980 by the pioneering work of Breslow and Rideout, who demonstrated that Diels–Alder reactions of water-soluble reagents would be greatly accelerated in aqueous solution [120]. In 2005, Sharpless et al. demonstrated that the Diels–Alder reaction of the water-insoluble reactants showed substantial rate acceleration in aqueous suspension over homogeneous solution [121, 122]. Some examples of domino Knoevenagel HDA reactions of 1-oxa-1,3-butadienes with intramolecular cycloaddition in water as the solvent are described below. Moghaddam et al. examined domino Knoevenagel HDA of 4-hydroxy-dithiocoumarin **194** and *O*-acrylated salicylaldehyde derivatives **193** in water (Scheme 35) [123]. Pentacyclic heterocycles **196** and **197** were formed by a catalyst-free method in good yields and with high regio- and stereo-selectivity.

Aldehydes **193** underwent the Knoevenagel condensation with 4-hydroxy-dithiocoumarin **194** in water at reflux to give the intermediates **195** in which two different heterodiene fragments were presented. The thiocarbonyl group of the thioester **195** reacted as heterodiene. The cycloadducts were obtained as a mixture of *cis*- and *trans*-isomers. The authors observed the influence of the substituent *R* ^*2*^ on reaction diastereoselectivity. The *trans*-isomer **196** was the main product for some reactions whereas for others, the products **197** were formed with the predominance of the *cis*-isomers (Scheme 35).

**Scheme 36:**
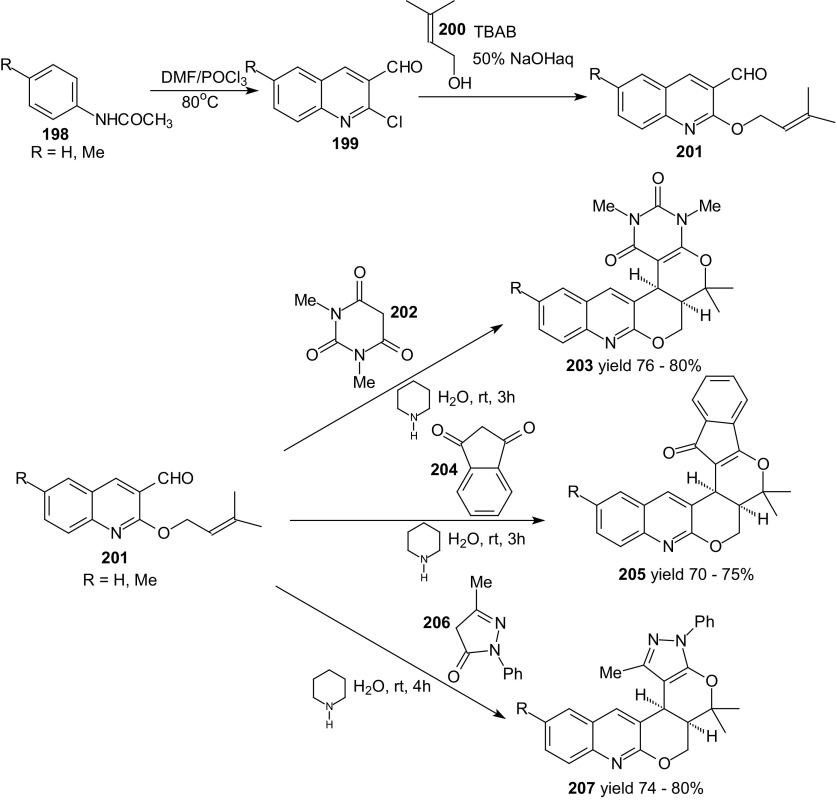
Synthesis of 3-formyl quinoline **201** containing dienophile moiety. Domino Knoevenagel HDA reactions of **201** and active methylene compounds **202**, **204** and **206**

The importance of quinoline and its fused derivatives prompted Baruah and Bhuyan to study the domino Knoevenagel intramolecular HDA reactions of 3-formyl quinoline containing a dienophile moiety [124]. Appropriate 1-oxa-1,3-butadienes were prepared from acetanilides **198** by treatment with Vilsmeier reagent (Scheme 36).

To introduce the dienophile in compound **201**, the reactions of 2-chloro-3-formylquinolines **199** with alcohol **200** in presence of aqueous sodium hydroxide under phase transfer catalytic conditions were used. Domino Knoevenagel HDA reactions of **201** and active methylene compound **202**, **204** and **206** in presence of piperidine at room temperature in water gave the *cis* fused penta or hexacyclic pyrano[2,3-*b*]quinoline derivative **203**, **205** or **207** with high yield (70–80 %) and diastereoselectivity (>99 %). The products were isolated by filtration in the pure form from aqueous medium.

**Scheme 37:**
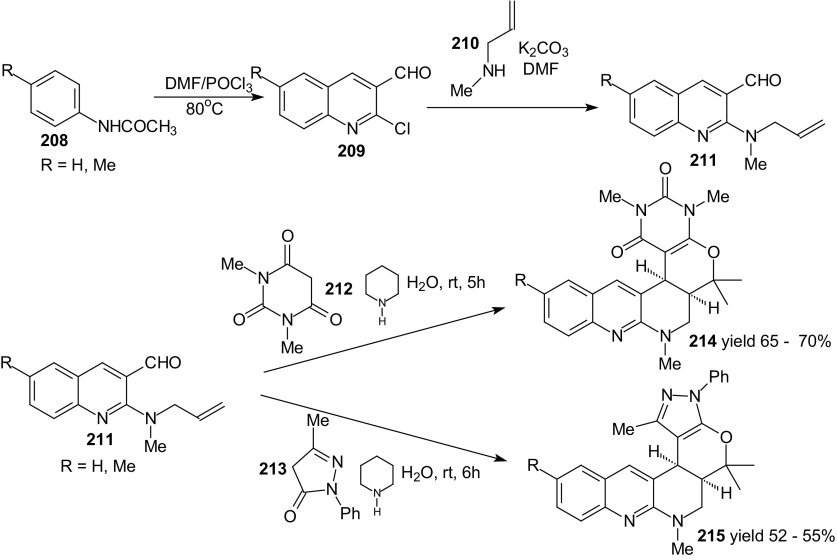
Synthesis of 3-formyl quinoline **211** containing dienophile moiety. Domino Knoevenagel HDA reactions of **211** and active methylene compounds **212** and **213**

Baruah and Bhuyan also studied the HDA reactions for other 1-oxa-1,3-butadienes (Scheme 37) [124]. These compounds possessing diene and dienophile moieties were prepared from aldehydes **209** by treatment with *N*-allyl methyl amine **210** in presence of K_2_CO_3_. Obtained products **211** on treatment with **212** or **213** in presence of piperidine in water at room temperature afforded the cycloadducts **214** or **215** in 52–70 % yields.

**Scheme 38:**
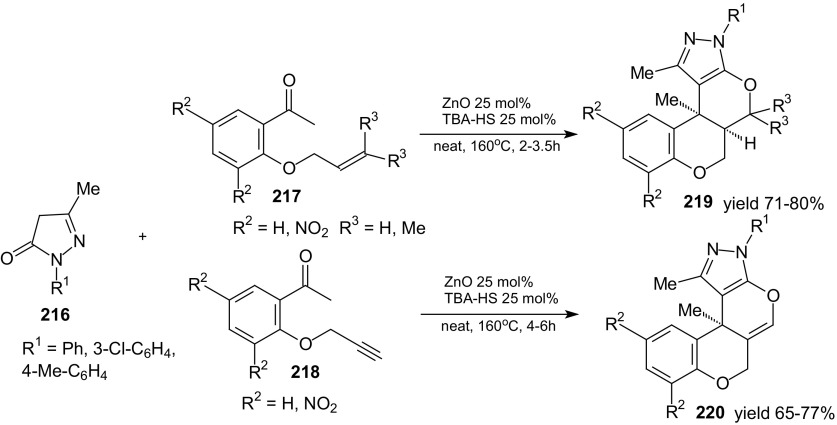
Domino Knoevenagel HDA reaction of 2-(alkenloxy)- and 2-(alkynyloxy)acetophenones **217** and **218** with pyrazolones **216**. Synthesis of chromeno-fused pyrazoles **219** and **220**

Parmar et al. used alkenyl- and alkynyl-ether-tethered ketones instead of aldehydes to extend the substrate scope in domino Knoevenagel intramolecular HDA reactions of 1-oxa-1,3-butadienes. They synthesized dihydropyran derivatives **219** and **220** as single stereoisomers with a tertiary ring junction carbon by solvent-free one-pot procedure in the presence of tetrabutylammonium-hydrogensulfate (Scheme 38) [125].

## Application of Inverse-Electron-Demand Hetero-Diels–Alder Reactions of 1-Oxa-1,3-Butadienes in Bioorthogonal Chemistry

**Scheme 39:**
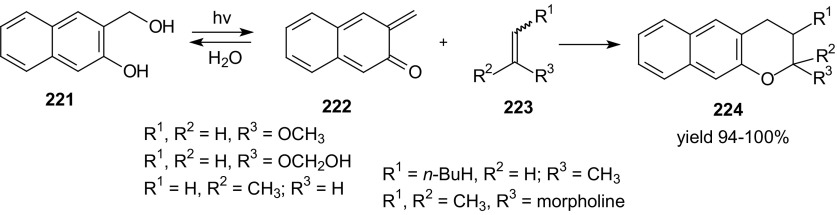
HDA cycloadditions of photochemically generated *o*-naphthoquinone methides **222** with vinyl ethers or enamines **223**

For chemical biologists, discovering new reactions which can expand the toolbox of bioorthogonal chemistry is a current challenge. Development of new orthogonal methods for labeling in the biosystems is still continued, although effective bioorthogonal reactions such as copper-free click chemistry have been developed [126]. Reactions which can be used in bioorthogonal click chemistry should meet the requirements: high reactivity and selectivity of reagent functional groups, chemical stability in aqueous solutions in vivo, biocompatibility and high reaction rate under physiological conditions [127–130]. Bioorthogonal ligations have been widely used in biomedical research since they can selective labels of biomolecules in living systems. Some of inverse-electron-demand HDA reactions 1-oxa-1,3-butadienes developed in recent years fulfill the criteria of click chemistry compiled by Sharpless [131, 132] and, in the future, can be used as bioorthogonal cycloaddition. There is only one example in the literature of application of inverse-electron-demand HDA reactions of 1-oxa-1,3-butadienes in bioorthogonal chemistry. Lei et al. described a new bioorthogonal ligation by click HDA cycloaddition of in situ-generated *o*-quinolinone quinone methides and vinyl thioethers [133]. High selectivity and the fact that this cycloaddition can proceed smoothly under aqueous conditions make it suitable for bioorthogonal chemistry. *o*-Quinone methides represent an 1-oxa-1,3-butadiene system which can undergo quick and selective inverse-electron-demand HDA cycloadditions. It is important that generation of the *o*-quinone methides can’t be conducted in harsh reaction conditions because it could be harmful for the organism cells. HDA cycloadditions of photochemically generated o-naphthoquinone methides **222** with vinyl ethers or enamines **223** as dienophiles were described by Arumugam and Popik (Scheme 39) [134–136]. They used ultraviolet (UV) light to generate 1-oxa-1,3-butadienes **222**.

**Scheme 40:**
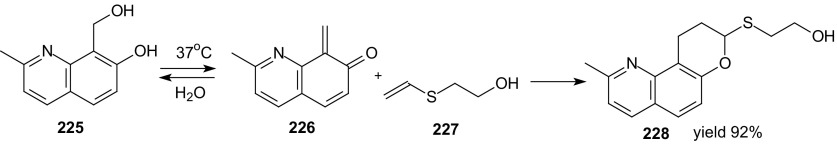
HDA cycloaddition of *o*-quinolinone quinine methide **226** and vinyl thioether **227** under physiological conditions

Li et al. optimized both reaction partners to make the reaction suitable for bioorthogonal ligation [133]. Introduction of more electronegative nitrogen into a heterodiene system **221** improved its reactivity and hydrophilicity (Scheme 40). As dienophile was used small and chemically stable in vivo vinyl thioether **227**. *o*-Quinolinone quinine methide **226** was prepared from 8-(hydroxymehyl)-2-methylquinolin-7-ol **225** without use of catalyst and UV light. Cycloreactants **226** and **227** underwent HDA cycloaddition under physiological conditions (37 °C, H_2_O). The authors used this bioorthonogal cycloaddition for labeling of proteins and imaging of taxol derivatives inside live cells.

**Scheme 41:**
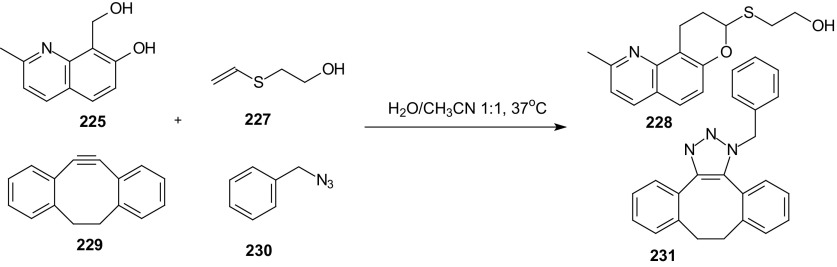
HDA reaction of **225** with **227** and SPAAC of **229** with **230** proceeded simultaneously without interfering with each other

Li et al. proved that HDA cycloaddition of *o*-quinolinone quinine methide **226** and vinyl thioether **227** can be utilized for labeling of multiple biomolecules in complex living systems when it is combined with other methods [137]. When cycloreactants **225** and **227** were combined with 5,6-didehydro-11,12-didehydrodibenzo[a,e]cyclooctene **229** and (azidomethyl)benzene **230** in a mixture of H_2_O/CH_3_CN at 37 °C, only products **228** and **231** were obtained, and no cross reaction products were prepared (Scheme 41). 1,3-Dipolar cycloaddition of azide **230** and alkyne **229** is widely used in bioorthogonal ligation as strain-promoted azide alkyne cycloaddition (SPAAC). The results indicated that these two ligations proceeded simultaneously without interfering with each other.

It seems that some of the HDA reactions described in Chapter 2 can be used in bioorthogonal chemistry in the future because they are selective, non-toxic, and can function in biological conditions taking into account pH, an aqueous environment, and temperature.

## Conclusion

This review article is an effort to summarize recent developments in inverse-electron-demand HDA reactions of 1-oxa-1,3-butadienes. Some of the papers related to the inverse-electron-demand HDA reactions of 1-oxa-1,3-butadienes found in the literature clearly demonstrate the importance of this transformation which opened up efficient and creative routes to different natural products containing six-membered oxygen ring systems. This type of cycloaddition is today one of the most important methods for the synthesis of dihydropyrans which are the key building blocks in carbohydrate derivative synthesis. Especially, the domino Knoevenagel HDA reactions have been frequently applied for the synthesis of natural products. The main advantage of the inverse-electron-demand HDA reaction of oxabutadienes is formation of dihydropyran derivatives with up to three stereogenic centers in one step from simple achiral precursors. This transformation characterizes the huge diversity, excellent efficiency, high regioselectivity, diastereoselectivity, and enantioselectivity observed in many cases. In recent years, the use of chiral Lewis acids, chiral organocatalysts, new heterodienes, or new dienophiles have given enormous progress. Recently, HDA reactions of 1-oxabutadienes conducted without a solvent or in water were developed and the results suggested that the presented green methods may displace other methods that use various organic solvents and that are performed at high temperature. Application of inverse-electron-demand HDA reactions of 1-oxa-1,3-butadienes in bioorthogonal chemistry is still challenging because there is only one example of this bioorthogonal cycloaddition in the literature. The author of this review sincerely hopes that this article will stimulate future research in bioorthogonal inverse-electron-demand cycloaddition of 1-oxa-1,3-butadienes and will encourage scientists to design novel bioorthogonal ligations.

## Abbreviations

Ac: Acetyl
*i*Bu: Isobutyl
*n*-Bu: *n*-Butyl
*t*-Bu: *tert*-Butyl
(S,S)- *t*-Bu-box: (*S,S*)-tert-Butylbis(oxazoline)
[bmim][NO_3_]: 1-Butyl-3-methylimidazolium nitrate
Bn: Benzyl
BPin: 3-Boronopinacol
Bz: Benzoyl
Cb: Benzyloxycarbonyl
DEA: Diethylamine
DIC: *N*,*N′*-Diisopropylcarbodiimide
DMAP: *N*,*N*-Ddimethyl-4-aminopyridine
DMF: Dimethylformamide
DMSO: Dimethyl sulfoxide
EDDA: Ethylene diammonium diacetate
Eu(fod)_3_: 6,6,7,7,8,8,8-Heptafluoro-2,2-dimethyl-3,5-octanedionato europium
IBX: *o*-Iodoxybenzoic acid
MDO: Modularly designed organocatalyst
MS: Molecular sieves
PCC: Pyridinium chlorochromate
PDC: Pyridinium dichromate
*i*Pr: Isopropyl
TBAB: tetra-*n*-Butylammonium bromide
TBAF: Tetrabutylammonium fluoride
TBA-HS: Tetrabutylammonium hydrogen sulfate
TBDMS: *tert*-Butyldimethylsilyl
TBDPS: *tert*-Butyldiphenylsilyl
TBS: *tert*-Butyldimethylsilyl
Tf: Trifluoromethanesulfonyl
THF: Tetrahydrofuran
